# Bio-Inspired Perception–Memory Coupling for Robust LiDAR–Inertial Odometry in Dynamic Environments

**DOI:** 10.3390/biomimetics11090624

**Published:** 2026-09-02

**Authors:** Bojia Hou, Fei Yu, Ya Zhang, Baojin Ping, Zhaoxu Wang

**Affiliations:** School of Instrument Science and Engineering, Harbin Institute of Technology, Harbin 150001, China; 25s001036@stu.hit.edu.cn (B.H.); yufei@hit.edu.cn (F.Y.); pingbaojin@stu.hit.edu.cn (B.P.); 24s101113@stu.hit.edu.cn (Z.W.)

**Keywords:** bio-inspired robotic perception, intelligent mobile robots, LiDAR–inertial odometry, multi-cue reliability, perception–memory coupling, dynamic environments, sensor fusion

## Abstract

Bionic intelligent robots operating in unstructured dynamic environments require perception systems that regulate uncertain observations according to their reliability and avoid converting transient disturbances into persistent spatial references. Inspired by reliability-weighted multisensory integration and by a functional abstraction of persistence-based evidence consolidation in biological navigation, this paper proposes a bio-inspired reliability-constrained LiDAR–inertial odometry framework. Each point-to-map observation is evaluated using residual consistency, local geometric quality, and voxel-level temporal stability. The fused reliability score regulates both the ESIKF state update and incremental map maintenance: low-confidence observations are continuously down-weighted, highly reliable points are admitted to the persistent map, ambiguous points are retained in short-term candidate memory for multi-frame verification, and unreliable points are rejected. The framework translates biological design principles into an engineering perception–memory architecture rather than reproducing a specific neural circuit. Repeated experiments on public datasets and a wheeled mobile robot platform show comparable accuracy in two normal sequences. Across five dynamic sequences, the complete method reduces localization RMSE by 14.08–28.88% relative to Fast-LIO2 and achieves lower mean RMSE than Dynamic-LIO on all five evaluated dynamic sequences. Frozen-map evaluation further yields 7.77–15.71% lower point-to-map RMSE together with higher consistent-correspondence ratios and coverage, while the maximum mean RMSE deviation in the parameter-sensitivity study remains below 7.2%. The maximum measured processing time is 18.69 ms per scan, maintaining real-time operation for a 10 Hz LiDAR.

## 1. Introduction

Bionic intelligent robots are expected to maintain autonomous navigation in complex, dynamic, and partially unstructured environments. Biological perceptual systems regulate the contribution of sensory cues according to their reliability, while navigation circuits integrate spatial cues with different levels of informativeness and learn their relationships over time [1,2]. These findings suggest a general design principle for robotic localization: when environmental conditions vary, observations should not be treated as equally trustworthy, and uncertain evidence should be prevented from exerting persistent influence on subsequent perception.

In robotic systems, accurate and continuous localization is a fundamental prerequisite for autonomous navigation in complex urban environments. For mobile robots and low-speed autonomous vehicles used in intelligent transportation, unmanned inspection, campus logistics, and autonomous driving, localization performance directly affects pose estimation, path planning, motion control, and map updating. Robust real-time localization is therefore essential for deploying autonomous systems beyond structured test sites and into real-world dynamic scenarios [3].

Current localization systems mainly rely on multi-sensor fusion. GNSS provides global positioning information, but it is vulnerable to multipath effects, signal attenuation, and outages in urban canyons, tunnels, tree-lined roads, and areas occluded by high-rise buildings [4]. IMU measurements provide high-rate motion information and short-term continuity, yet stand-alone inertial navigation suffers from cumulative drift. Vision sensors offer rich texture and semantic cues, but their performance is easily degraded by illumination changes, motion blur, and weak-texture conditions. By contrast, LiDAR provides active sensing and accurate three-dimensional geometric measurements. When tightly coupled with IMU, LiDAR–IMU fusion combines stable geometric constraints with high-rate motion propagation, making it an important solution for real-time localization [5,6].

Among LiDAR–IMU methods, LOAM established a representative framework for three-dimensional LiDAR odometry and mapping using edge and planar features. However, its front end depends on hand-crafted feature extraction and is sensitive to environmental structure [5]. LIO-SAM further incorporated LiDAR, IMU, and loop-closure constraints into a factor-graph framework, improving consistency and global correction capability, but its smoothing-based formulation increases computational cost and real-time pressure [6]. The tightly coupled 3D LiDAR–IMU odometry proposed by Ye et al. strengthens the complementarity between inertial propagation and LiDAR matching, but its correction still depends mainly on stable geometric correspondences [7]. FAST-LIO and Fast-LIO2 further improved real-time performance by using efficient iterated filtering and direct point-to-map updates with incremental mapping [8,9]. Nevertheless, these methods still rely heavily on the assumption that the local map and point-to-map correspondences are reliable. Faster-LIO mainly improves front-end efficiency [10], whereas VoxelMap improves map representation [11]. These advances do not explicitly answer a key dynamic-scene question: how should the same unreliable observation be treated when it can simultaneously affect the current state update and the long-term incremental map?

This limitation becomes critical in urban environments, where vehicles, pedestrians, cyclists, moving vegetation, and temporary occlusions continuously disturb LiDAR observations. For direct point-to-map LIO, dynamic interference causes a coupled failure chain. First, corrupted point-to-plane correspondences bias the current state correction. Second, if these observations are inserted into the incremental map, they form spurious structures or ghost points. Third, the contaminated map further affects later nearest-neighbor search, plane fitting, and residual construction. Therefore, robustness in dynamic scenes cannot be obtained only by weakening outliers in the current frame; it must also control whether uncertain observations are allowed to become future map references.

Motivated by reliability-dependent cue regulation and staged handling of uncertain evidence, this paper proposes a bio-inspired reliability-constrained LiDAR–inertial odometry framework. The method is a functional algorithmic abstraction rather than a biologically faithful neural model. Residual consistency, local geometric quality, and voxel-level temporal stability are treated as complementary perceptual cues. Their fused reliability score jointly regulates current state estimation and the admission of observations into the incremental map. Low-confidence measurements are down-weighted; highly reliable points are admitted to persistent spatial representation; ambiguous points are retained in short-term candidate memory for temporal verification; and low-reliability points are rejected. This perception–memory coupling suppresses both the immediate influence of unreliable observations and their delayed propagation through map contamination while preserving the real-time structure of Fast-LIO2.

The main contributions of this paper are summarized as follows:

1. Inspired by reliability-dependent cue integration in biological perception, a multi-cue spatiotemporal reliability model is developed for LiDAR–inertial robotic localization. Residual consistency, local geometric quality, and voxel-level temporal stability are integrated to assess the usability of each point-to-map observation.
2. A functionally bio-inspired perception–memory coupling mechanism is proposed. The same reliability score regulates both the information contribution of an observation during ESIKF correction and its eligibility to become a persistent element of the spatial map.
3. A staged evidence-retention and delayed-consolidation strategy is designed for ambiguous observations. Multi-frame verification enables persistent static structures to be retained while reducing the probability that transient disturbances are incorporated into the incremental map.
4. The framework is validated through repeated cross-sequence experiments, comparison with Dynamic-LIO, frozen-map quality evaluation, hyperparameter sensitivity analysis, and runtime measurements on public datasets and a physical wheeled mobile robot.

The remainder of this paper is organized as follows. Section 2 reviews bio-inspired multisensory navigation, LiDAR–inertial odometry, dynamic-scene robust localization, and reliability-aware estimation. Section 3 presents the biological inspiration, multi-cue reliability modeling, reliability-gated state correction, and spatial-memory maintenance. Section 4 reports representative localization results, cross-sequence and external-method comparisons, persistent-map quality, hyperparameter sensitivity, ablation, and runtime analyses. Section 5 concludes the paper.

## 2. Related Work

### 2.1. Bio-Inspired Multisensory Navigation and LiDAR–Inertial Odometry

Biological navigation relies on integrating multiple sensory cues whose informativeness changes with environmental conditions. Neural and behavioral studies have shown that more reliable cues receive greater weight during multisensory estimation and that spatial navigation systems can learn relationships among cues with different levels of informativeness [1,2]. These studies directly support reliability-dependent cue regulation and multi-cue learning. The short-term candidate storage and persistent map construction used in this paper are functional robotic abstractions developed from these principles, rather than claims that the algorithm reproduces a specific neural memory system.

Within robotic localization, direct point-to-map LIO has become an important technical route for real-time localization because it avoids heavy feature engineering while maintaining high efficiency. FAST-LIO achieves high-frequency tightly coupled LiDAR–IMU state estimation using an iterated error-state Kalman filter [8]. Fast-LIO2 further removes explicit feature extraction and directly uses raw point-to-map constraints together with ikd-Tree-based incremental mapping, making it a representative direct LIO framework [9]. Faster-LIO improves front-end efficiency through a parallel sparse incremental voxel structure [10], while VoxelMap improves map representation using probabilistic adaptive voxels [11]. Point-LIO [12] further targets high-bandwidth LiDAR–inertial estimation, while R^2^LIVE [13] and R3LIVE [14] extend tightly coupled estimation toward LiDAR–inertial–visual sensing. However, these methods mainly focus on computational efficiency, sensing bandwidth, multimodal estimation, or map organization. In dynamic scenes, they still rely on the implicit assumption that local maps and point-to-map correspondences are dominated by static and reliable structures. They do not explicitly determine whether low-confidence measurements should influence the current update or whether such measurements should be retained in the long-term map.

### 2.2. Robust Localization in Dynamic Environments

Dynamic environments violate the static-world assumption of conventional SLAM and mapping. Existing methods usually suppress dynamic interference through semantic segmentation, object recognition, visibility analysis, occupancy change, or temporal consistency. Representative methods include SuMa++, DynaSLAM, Remove-then-Revert, ERASOR, MapCleaner, InsMOS, and ERASOR2 [15,16,17,18,19,20,21]. More recently, DRR-LIO and DO-Removal have addressed dynamic interference more directly in the LiDAR–inertial odometry front end [22,23]. SemanticKITTI [24] has also supported learning-based dynamic-scene understanding, including sequential moving-object segmentation methods [25]. Dynamic-LIO [26] uses label-consistency detection to identify and remove moving-object points within a LiDAR–inertial odometry framework. These studies demonstrate that moving objects and transient structures can degrade both localization and mapping. However, most of them focus on explicit dynamic-object detection or removal. They do not treat observation reliability as a continuous signal that jointly constrains both state estimation and incremental map maintenance.

### 2.3. Reliability-Aware State Estimation and Map Maintenance

Robust estimation weakens the influence of outliers by modifying residual weights or measurement covariance. In ICP and point-to-plane registration, this idea is reflected in assigning different confidence levels to different correspondences [27,28,29]. Global and robust registration methods such as Go-ICP, robust registration under extreme outlier rates, TEASER++, and graduated non-convexity further show that stable registration under high outlier rates depends on effective screening of poor correspondences [30,31,32,33]. Nevertheless, these methods mainly improve the robustness of the current registration or update step. They usually do not address whether a low-confidence measurement should continue to exist as a persistent element in an online incremental map.

Taken together, the works most directly related to this study are Fast-LIO2 and its map-maintenance variants [9,10,11], dynamic-scene suppression methods [15,16,17,18,19,20,21,22,23], and reliability-aware robust estimation methods [27,28,29,30,31,32,33]. Different from these studies, the proposed method is functionally inspired by reliability-dependent cue integration and staged evidence management. It uses a point-wise spatiotemporal reliability score to jointly regulate perceptual state correction and persistent map construction, addressing both instantaneous estimation robustness and the long-term propagation of map contamination in direct point-to-map LIO. The biological inspiration defines the functional architecture, whereas the Cauchy weighting, geometric statistics, temporal variance, and map-admission rules remain engineering realizations.

## 3. Proposed Method

### 3.1. Overall Framework

The proposed framework is motivated by reliability-dependent sensory integration observed in biological navigation [1,2] and by a functional engineering abstraction in which persistent evidence is preferentially retained in a stable spatial representation. The first principle is directly grounded in biological studies of cue weighting and learning; the second is used here as an algorithmic design analogy rather than as a claim of neural-mechanism reproduction.

This study translates these principles into four algorithmic operations: (i) residual, geometric, and temporal cues provide complementary evidence of observation reliability; (ii) the fused score regulates perceptual gain in the ESIKF update; (iii) ambiguous evidence is retained in a short-term candidate buffer; and (iv) only persistent evidence is promoted to the incremental map, while rejection and lifetime expiration suppress unstable observations. The candidate buffer therefore functions as temporary evidence storage and the incremental map as persistent spatial representation. These correspondences define the bio-inspired design logic, but they are not intended as one-to-one models of biological neural structures.

In direct point-to-map LIO, dynamic interference propagates through two coupled paths. First, unreliable point-to-plane residuals enter the iterative ESIKF update and may immediately bias perceptual state correction. Second, if the corresponding points are inserted into the incremental map, they become persistent spatial-memory elements and can later disturb nearest-neighbor search, local plane fitting, and residual construction. Dynamic-object-removal methods usually treat suppression as a preprocessing or binary classification problem, whereas the proposed framework uses a continuous reliability score to couple current perception with subsequent map-memory consolidation.

**Figure 1:**
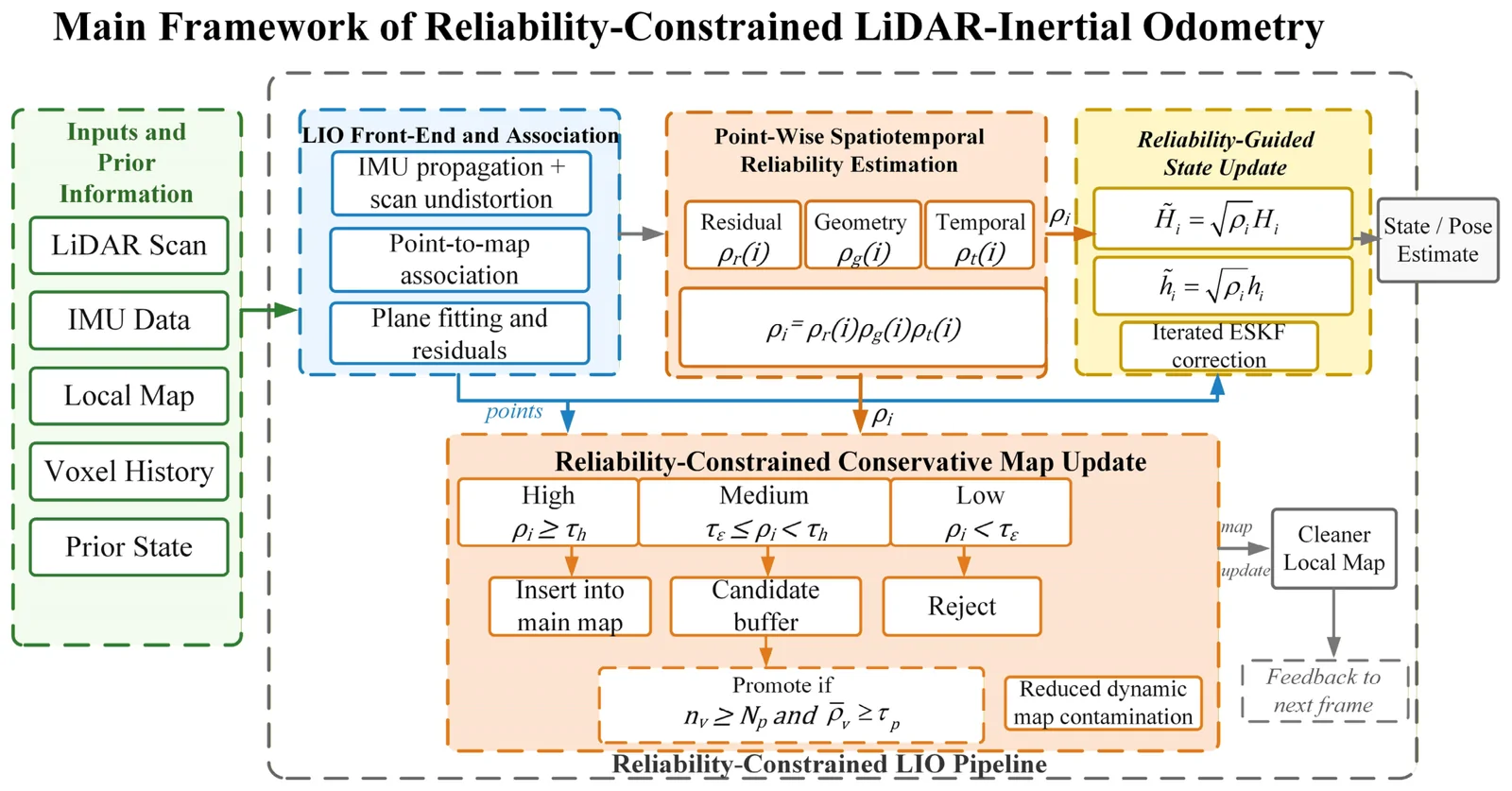
Overall framework of the proposed bio-inspired perception–memory coupling system. IMU propagation and LiDAR scan undistortion generate a motion-compensated scan; point-to-map association produces residuals; multi-cue reliability estimation regulates the state update; and the same reliability score governs the consolidation of observations into the incremental spatial map.

As shown in Figure 1, the baseline LIO pipeline is preserved, including IMU propagation, LiDAR scan undistortion, point-to-map association, and iterative state update. The reliability module is placed after point-to-map association because the residual value, local geometric support, and voxel-level temporal history are all available at this stage. The estimated point-wise reliability score is then sent to two branches: the reliability-regulated perception branch, where it adjusts the contribution of each observation, and the spatial-memory branch, where it determines whether the point is consolidated, retained temporarily, or rejected.

This design follows the coupled nature of the dynamic-scene failure mechanism. If reliability is used only by the estimator, a down-weighted but unstable observation may still enter the map and affect subsequent frames. If reliability is used only by the map module, the current filtering update remains vulnerable to biased residuals. The core idea is therefore to establish a consistent perception–memory rule: observations with weak evidence should have limited influence on the current correction and should not become persistent map references without multi-frame verification.

Accordingly, Section 3.2 focuses on bio-inspired multi-cue reliability modeling and reliability-gated perceptual state correction. Section 3.3 presents reliability-constrained spatial-memory maintenance, in which uncertain observations are retained in short-term candidate memory before being consolidated into the persistent map.

### 3.2. Bio-Inspired Multi-Cue Reliability and State Update

#### 3.2.1. Point-to-Map Observation Model

Let *p_i_* denote a LiDAR point in the current scan, and let $p_{i}^{W}$ denote the corresponding point transformed into the world coordinate system using the current state estimate. A local plane is fitted using neighboring map points, and its parameters are represented by the unit normal vector $n_{i}$ and distance term $d_{i}$. The point-to-plane residual is then written as

(1) $$r_{i}=n_{i}^{T}p_{i}^{W}+d_{i}$$

This residual is the basic observation used by direct point-to-map LIO for state correction. Since it is a standard component of the baseline filtering framework, this paper does not modify the residual model itself; instead, it modifies how each residual is weighted and whether the corresponding point is allowed to contribute to the incremental map.

Residual magnitude alone is insufficient for reliability judgment. A moving object may temporarily produce a small residual if it is close to an existing map plane, and a geometrically weak neighborhood may also yield a small residual while providing poor constraint information. Conversely, a newly observed static structure may initially lack temporal evidence. Therefore, the following reliability model evaluates observations using current residual evidence, local geometric evidence, and cross-frame temporal evidence.

#### 3.2.2. Multi-Cue Spatiotemporal Reliability

This subsection evaluates point-to-map observation usability rather than performing binary static/dynamic classification. Two existing routes are considered first. Residual-based robust LIO front ends mainly rely on single-frame residual magnitude; they can attenuate large residuals but cannot judge whether a small-residual observation has valid local geometry or remains stable over time. Front-end dynamic-region or object-removal methods usually treat dynamic suppression as a preprocessing binary decision; they reduce obvious dynamic interference but do not provide continuous confidence for each residual, nor do they link estimation weighting with map admission.

**Figure 2:**
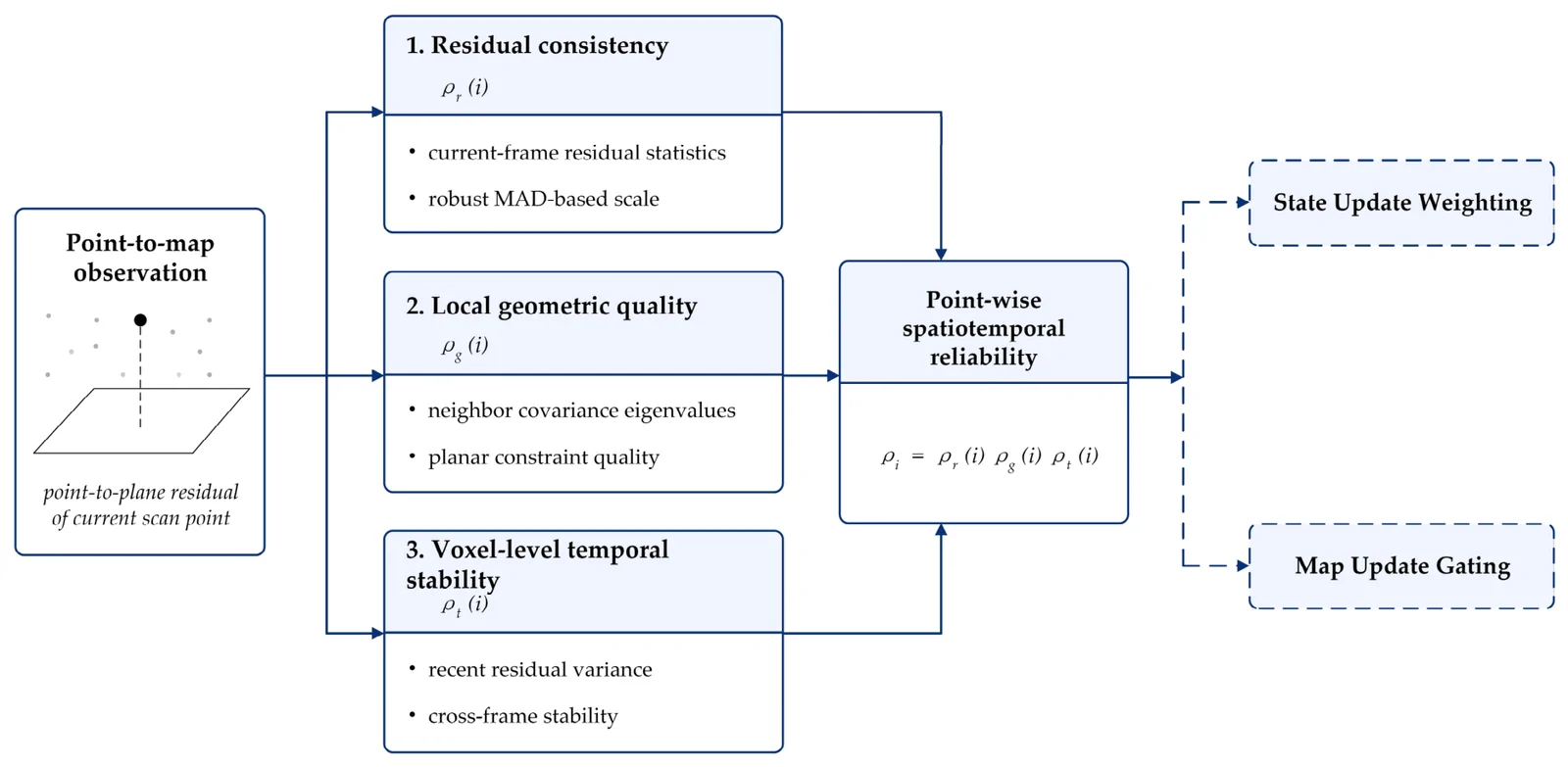
Multi-cue spatiotemporal reliability estimation. Residual consistency, local geometric quality, and voxel-level temporal stability jointly determine the reliability of each point-to-map observation and regulate both perceptual state correction and spatial-memory update.

To address these limitations, the proposed bio-inspired multi-cue model uses three complementary evidence sources: residual consistency for current-frame statistical abnormality, local geometric quality for constraint validity, and voxel-level temporal stability for cross-frame consistency. The resulting reliability score is reused by both the state update and map-memory modules, so that unreliable observations are down-weighted during filtering and further checked before entering persistent spatial memory. Figure 2 illustrates this shared reliability pathway.

The final reliability of the i-th observation is obtained by multiplying the three evidence terms:

(2) $$\rho_{i}=\rho_{r}(i)\rho_{g}(i)\rho_{t}(i)$$

where $\rho_{r}(i)$, $\rho_{g}(i)$, $\rho_{t}(i)$, and $\rho_{i}$ denote residual-consistency, geometric, temporal, and fused reliability, respectively. The product provides a conservative, non-compensatory aggregation of the three normalized evidence terms. If residual consistency, geometric validity, or temporal stability fails, the final score is rapidly reduced rather than being offset by a high score from another cue. This conservative behavior is suitable for map-memory consolidation, where admitting an unreliable point may create a persistent false reference for later associations.

(3) $$\rho_{i}\leq min\;\left\{\rho_{r}\left(i\right),\rho_{g}\left(i\right),\rho_{t}\left(i\right)\right\},\;\;\underset{\rho_{q}\left(i\right)\to 0}{lim}\rho_{i}=0,\;q\in \{r,g,t\}$$

More specifically, the three terms are treated as normalized evidence-support scores rather than as strictly independent probabilities. Their multiplication corresponds to the product t-norm, that is, a continuous soft-AND operation. Equation (3) shows that the fused reliability cannot exceed any individual evidence term and approaches zero when one cue becomes invalid. In contrast to an additive rule, a high score from one cue therefore cannot fully compensate for a near-failure of another cue. This non-compensatory property is consistent with the asymmetric risk of online map admission: temporarily withholding a marginal static point mainly reduces local map completeness, whereas admitting an unreliable point may create a persistent false reference for subsequent associations. From a bio-inspired perspective, the rule operationalizes the general principle that evidence with low trustworthiness should exert less influence on the current percept and should be less likely to enter a persistent spatial representation. The product t-norm is therefore an engineering realization of conservative cue integration, not a direct model of a specific neural computation or an assumption that the residual, geometric, and temporal evidence sources are statistically independent. Because each component score lies in the interval (0, 1], the fused reliability also satisfies 0 < ρ_i_ ≤ 1, and its maximum value is one only when all three component scores equal one. No single cue is assumed to be globally dominant. Under the product t-norm, the smallest component score acts as the limiting factor for a given observation, so the dominant limiting cue can change with the current residual, geometry, and temporal history.

Residual consistency is the first evidence source. It measures whether the current point-to-plane residual deviates from the residual distribution of the frame. A large deviation usually indicates poor association, dynamic interference, or local map inconsistency. The residual reliability is defined by a Cauchy-type function:

(4) $$\rho_{r}(i)=\frac{c^{2}}{c^{2}+r_{i}^{2}}$$

where

(5) $$c=k_{r}\sigma_{r}$$

Here, $r_{i}$ (m) is the signed point-to-plane residual of the i-th current-scan point, $\sigma_{r}$ (m) is the robust residual scale estimated from all valid residuals in the current scan, $\kappa_{r}$ is a dimensionless scale coefficient, and c (m) is the resulting Cauchy scale. The robust scale is computed using the median absolute deviation (MAD):

(6) $$\sigma_{r}=1.4826\;{median}_{i}\;\left(\left|r_{i}-{median}_{j}\left(r_{j}\right)\right|\right)$$

The factor 1.4826 makes the MAD a consistent estimate of the standard deviation for Gaussian inliers. The coefficient $\kappa_{r}$ controls the relative attenuation range, while $c=\kappa_{r}\sigma_{r}$ adapts the Cauchy kernel to the residual dispersion of each scan. Accordingly, the value of $\rho_{r}\left(i\right)$ is bounded between zero and one, with one included. It equals one at $r_{i}=0$ and decreases smoothly as $\left|r_{i}\right|$ increases; it reaches 0.5 when $\left|r_{i}\right|=c$.

The scale is estimated from the current residual distribution using MAD-based robust statistics. The Cauchy-type function gives smooth attenuation: it suppresses large residuals more continuously than Huber weighting and avoids the abrupt zero-weight cutoff of Tukey-type rejection. This matches the proposed strategy of first weakening uncertain observations and then verifying them through map-side delayed confirmation, rather than discarding potentially useful static structures too early. However, residual evidence is still frame-local, so it must be complemented by geometric evidence.

Geometric reliability is the second evidence source. Its purpose is to evaluate whether the neighboring map points form a valid local planar constraint, which is usually ignored by residual-only robust kernels. A small residual from a neighborhood with excessive normal-direction scatter or weak planar support may still be unsafe for state correction and map insertion. For the N_i_ neighboring map points used in plane fitting, the 3 × 3 covariance matrix is computed as $C_{i}=(1/N_{i})\Sigma_{j}\left(p_{j}-\bar{p}\right){\left(p_{j}-\bar{p}\right)}^{T}$. Let λ_1_ ≤ λ_2_ ≤ λ_3_ denote the eigenvalues of C_i_, where λ_1_ corresponds to the fitted plane-normal direction. Using these covariance eigenvalues, the local structural uncertainty is defined as

(7) $$\eta_{i}=\frac{\lambda_{1}}{\lambda_{1}+\lambda_{2}+\lambda_{3}+\varepsilon }$$

The corresponding geometric reliability is then computed as

(8) $$\rho_{g}(i)=\exp\left(-\frac{\eta_{i}}{\sigma_{g}}\right)$$

In (7) and (8), $\lambda_{1}$ denotes the eigenvalue associated with the fitted plane-normal direction, i.e., the smallest eigenvalue of the covariance matrix of the neighboring map points, while $\lambda_{2}$ and $\lambda_{3}$ denote the two tangential-direction eigenvalues. The dimensionless quantity $\eta_{i}$ measures the proportion of local scatter along the normal direction; a smaller $\eta_{i}$ indicates a better-supported planar constraint. The constant ε>0 prevents numerical division by zero, and $\sigma_{g}>0$ is the geometric attenuation scale controlling how rapidly $\rho_{g}\left(i\right)$ decreases with $\eta_{i}$. Therefore, the value of $\rho_{g}\left(i\right)$ is bounded between zero and one, with values close to one for well-supported planar neighborhoods and smaller values for geometrically uncertain neighborhoods.

A larger $\eta_{i}$ leads to lower geometric reliability. This term prevents poorly constrained local neighborhoods from contributing like well-supported planar regions. Its value is not merely plane-quality description, but a constraint-validity test that complements residual weighting: residual consistency suppresses observations that disagree with the map, whereas geometric reliability suppresses observations whose residuals look acceptable but whose local constraints are structurally unreliable. Since both cues are still instantaneous, temporal evidence is further required.

Temporal reliability is the third evidence source. For each voxel, recent residual statistics are maintained to evaluate whether observations from the same spatial region remain stable over time:

(9) $$\rho_{t}(i)=\exp\left(-\frac{s_{k}^{2}}{\sigma_{t}^{2}+\varepsilon }\right)$$

In (9), $v\left(i\right)$ denotes the voxel containing the i-th observation, and $s_{v\left(i\right)}^{2}$, with units of m^2^, is the variance of the recent point-to-plane residuals maintained for that voxel. The parameter $\sigma_{t}^{2}>0$, also with units of m^2^, is the temporal variance scale used to normalize residual fluctuation, and ε is the same positive regularization constant used to avoid a zero denominator. Thus, the value of $\rho_{t}\left(i\right)$ is bounded between zero and one. A voxel with stable residual history yields a value close to one, whereas increasing cross-frame residual variance produces exponential attenuation.

Large residual fluctuations indicate temporal instability and reduce voxel reliability. Voxel-level residual variance is used because it directly measures the stability of the quantity used in state correction. Occupancy probability mainly indicates whether a voxel has been observed, and point density is strongly affected by sampling and viewpoint; both can be high for static structures and dynamic objects. In contrast, residual variance evaluates whether the region repeatedly provides stable registration constraints, while remaining lightweight because it avoids point-wise history storage and semantic labels.

Overall, the three terms form a progressive reliability test: residual consistency detects abnormal associations, geometric reliability verifies constraint validity, and temporal reliability checks cross-frame stability. The fused score is then shared by state update and map maintenance, which distinguishes the proposed method from single-frame residual reweighting or front-end binary dynamic removal.

Although several quantities appear in the reliability model, they do not all represent manually tuned hyperparameters. The residual scale *σ_r_* is estimated online from the current-frame residual distribution using robust MAD statistics. The local geometric uncertainty *η_i_* is directly calculated from the normalized covariance eigenvalues of the neighboring map points, while the temporal statistic *s_ν_*^2^ is updated online from the recent residual history of the corresponding voxel. Therefore, *σ_r_*, *η_i_*, and *s_ν_*^2^ are data-dependent statistics rather than free parameters. The manually specified quantities are limited to a small number of dimensionless scale coefficients and map-decision settings.

#### 3.2.3. Reliability-Gated Perceptual State Correction

After obtaining ρ_i_, the reliability score is used as a continuous perceptual gain in the filtering update. Let *H_i_* denote the Jacobian matrix of the corresponding point-to-plane observation, and let *h_i_* denote the associated residual term. The reliability-weighted observation is written as

(10) $${\tilde{H}}_{i}=\sqrt{\rho_{i}}H_{i}$$

(11) $${\tilde{h}}_{i}=\sqrt{\rho_{i}}h_{i}$$

Using $\sqrt{\rho_{i}}$ to rescale both the Jacobian and the residual is equivalent to reducing the information contribution of low-reliability observations, or equivalently inflating their effective measurement covariance. High-reliability observations retain their original influence, whereas low-confidence observations are weakened during state correction instead of being removed by a hard threshold.

The square-root form can be derived directly from the weighted ESIKF measurement update. After linearization, the stacked point-to-map residual model is written as

(12) $$h=H\;\delta x+v,\;\;v∼N\left(0,R\right)$$

where h stacks the point-to-plane residual terms, H is the corresponding Jacobian matrix, δx is the error-state increment, and R is the measurement-noise covariance. Let $W=diag\left(\rho_{1},\ldots ,\rho_{m}\right)$ be the diagonal reliability matrix. The reliability-weighted maximum a posteriori objective can then be expressed as

(13) $$J\left(\delta x\right)={∥\delta x∥}_{P^{-1}}^{2}+{∥W^{1/2}\;\left(h-H\delta x\right)∥}_{R^{-1}}^{2}$$

where P is the prior error-state covariance and ${∥a∥}_{Q}^{2}=a^{T}Qa$. Equation (13) is implemented by transforming the measurement system as

(14) $$\tilde{H}=W^{1/2}H,\;\;\tilde{h}=W^{1/2}h$$

For the i-th observation, the corresponding contribution to the measurement information matrix becomes

(15) $${\tilde{H}}_{i}^{T}R_{i}^{-1}{\tilde{H}}_{i}=\rho_{i}\;H_{i}^{T}R_{i}^{-1}H_{i}$$

Therefore, multiplying both $H_{i}$ and $h_{i}$ by $\sqrt{\rho_{i}}$ makes $\rho_{i}$ scale the observation information linearly. Directly multiplying them by $\rho_{i}$ would instead produce a quadratic information factor $\rho_{i}^{2}$ and would over-attenuate low-reliability observations. For scalar or independently modeled point-to-plane observations, the same operation is equivalently interpreted as inflating the effective measurement covariance:

(16) $$R_{i,eff}=\frac{R_{i}}{\rho_{i}}$$

Hence, a high-reliability observation retains approximately its nominal covariance, while a low-reliability observation is assigned a larger effective covariance and contributes less to the Kalman correction. This derivation explains both the role of $\sqrt{\rho_{i}}$ in (10) and (11) and the mechanism through which the reliability score affects the ESIKF update.

This forms the perceptual role of the reliability score: suppressing the instantaneous influence of unreliable observations on the current state update. Continuous weighting is less aggressive than hard rejection, which is useful because medium-reliability points may still contain valid geometric information and should not be discarded prematurely. However, perceptual weighting alone does not determine whether a weakened observation can enter persistent spatial memory. Once inserted into the incremental map, it may become a false reference and continue to affect subsequent point-to-map associations. Therefore, limiting only the current-frame influence is insufficient, and the same reliability score must also govern map-memory consolidation.

### 3.3. Reliability-Constrained Spatial-Memory Maintenance

#### 3.3.1. Three-Level Map-Memory Update Strategy

Reliability weighting suppresses the instantaneous influence of unreliable observations, but it does not determine whether these observations are allowed to become long-term spatial-memory references. In many direct LIO implementations, motion-compensated and downsampled scan points are inserted into the incremental map once they pass basic geometric or spatial filtering. Existing dynamic-object-removal or map-cleaning methods often detect or remove dynamic points before estimation or clean an already built map afterward. The proposed module instead uses the same reliability score that regulates perception to determine whether a point is consolidated into persistent spatial memory, retained temporarily, or rejected.

(17) $$p_{i}\to M,\;\;\;\;\mathrm{if}\;\rho_{i}\geq \tau_{h}$$

(18) $$p_{i}\to C,\;\;\;\;\mathrm{if}\;\tau_{l}\leq \rho_{i}<\tau_{h}$$

(19) $$p_{i}\to \emptyset ,\;\;\;\;\mathrm{if}\;\rho_{i}<\tau_{l}$$

where M denotes the persistent incremental map memory, C denotes the short-term candidate-memory buffer, and τ_l_ and τ_h_ denote the low- and high-reliability thresholds, respectively. In all experiments, the two thresholds are uniformly fixed at τ_l_ = 0.35 and τ_h_ = 0.65.

Rather than treating them as two independently tuned parameters, the thresholds are symmetrically parameterized around the neutral reliability level of 0.5 as *τ_l_* = 0.5 − Δ*τ* and *τ_h_* = 0.5 + Δ*τ*, where the decision-band half-width is fixed at Δ*τ* = 0.15. Therefore, the low- and high-reliability thresholds introduce only one independent decision parameter. The resulting interval defines a three-way reliability decision: observations above the upper boundary are accepted, observations below the lower boundary are rejected, and observations within the intermediate interval are assigned to delayed confirmation.

The static-dominant calibration segment is used only to verify that the selected decision band provides a reasonable separation among reliable, ambiguous, and clearly unstable observations; it is not used to optimize the thresholds separately for individual test sequences. Moreover, the thresholds operate on the fused dimensionless reliability score rather than on raw residual magnitude, point number, or point-cloud density. Their numerical meanings are therefore not directly tied to a particular scene scale.

The value of this three-level strategy lies in replacing one-shot map insertion with reliability-driven decision making. Conventional direct insertion tends to favor map completeness but risks storing transient dynamic points, whereas hard removal tends to preserve map purity but may discard newly observed static structures. The proposed thresholds define a quantitative compromise between these two objectives: high-reliability observations are inserted immediately to maintain map growth, low-reliability observations are rejected to protect map purity, and medium-reliability observations are temporarily retained for delayed confirmation. Functionally, this three-way process can be interpreted as staged evidence management: immediate acceptance corresponds to high-confidence admission, temporary buffering corresponds to short-term retention, and rejection corresponds to inhibition of unstable evidence. This interpretation clarifies the bio-inspired design intent but does not imply a biologically faithful model of memory consolidation.

#### 3.3.2. Short-Term Candidate Memory and Delayed Consolidation

Medium-reliability observations are retained in short-term candidate memory because they may correspond either to newly observed static structures or to transient dynamic interference. For each candidate voxel, the system maintains a stable observation count and an average reliability. A candidate observation is consolidated into the persistent map only when the following conditions are satisfied:

(20) $$n_{v}\geq N_{p}$$

(21) $$\;{\bar{\rho }}_{v}\geq \tau_{p}$$

The candidate-promotion reliability threshold is not independently tuned. It is defined as the midpoint of the three-way decision interval, *τ_p_* = (*τ_l_* + *τ_h_*)/2 = 0.50, and therefore does not introduce an additional degree of freedom. The promotion count *N_p_* = 2 represents a two-scan persistence requirement in the 10 Hz implementation rather than a sequence-specific accuracy parameter. Its purpose is to prevent a candidate from being promoted on the basis of a single transient observation.

When these conditions are satisfied, the candidate observation is consolidated into the persistent map; otherwise, it remains in short-term memory until its lifetime expires. This delayed-consolidation mechanism is introduced because medium-reliability observations cannot be handled reliably by a binary keep-or-remove rule. They may correspond to new static structures whose evidence has not yet accumulated, or to transient dynamic points that should not become persistent references. Multi-frame verification allows uncertain observations to accumulate evidence before long-term map admission, thereby preserving map completeness while reducing the consolidation of transient interference. In this paper, “consolidation” specifically denotes the algorithmic promotion of repeatedly supported observations into the persistent map and does not imply a biologically faithful neural consolidation process.

#### 3.3.3. Operation Rules for Spatial-Memory Maintenance

In implementation, candidate observations are assigned a maximum lifetime. If a candidate does not satisfy the consolidation conditions within this lifetime, it is removed from short-term memory.

The operation rules separate three decisions that are usually coupled in direct map insertion: whether an observation should influence the current state, whether it should be retained temporarily for verification, and whether it is qualified to become a persistent spatial-memory reference. These decisions are all driven by the same reliability score, which keeps the perception and map-memory modules logically consistent.

**Figure 3:**
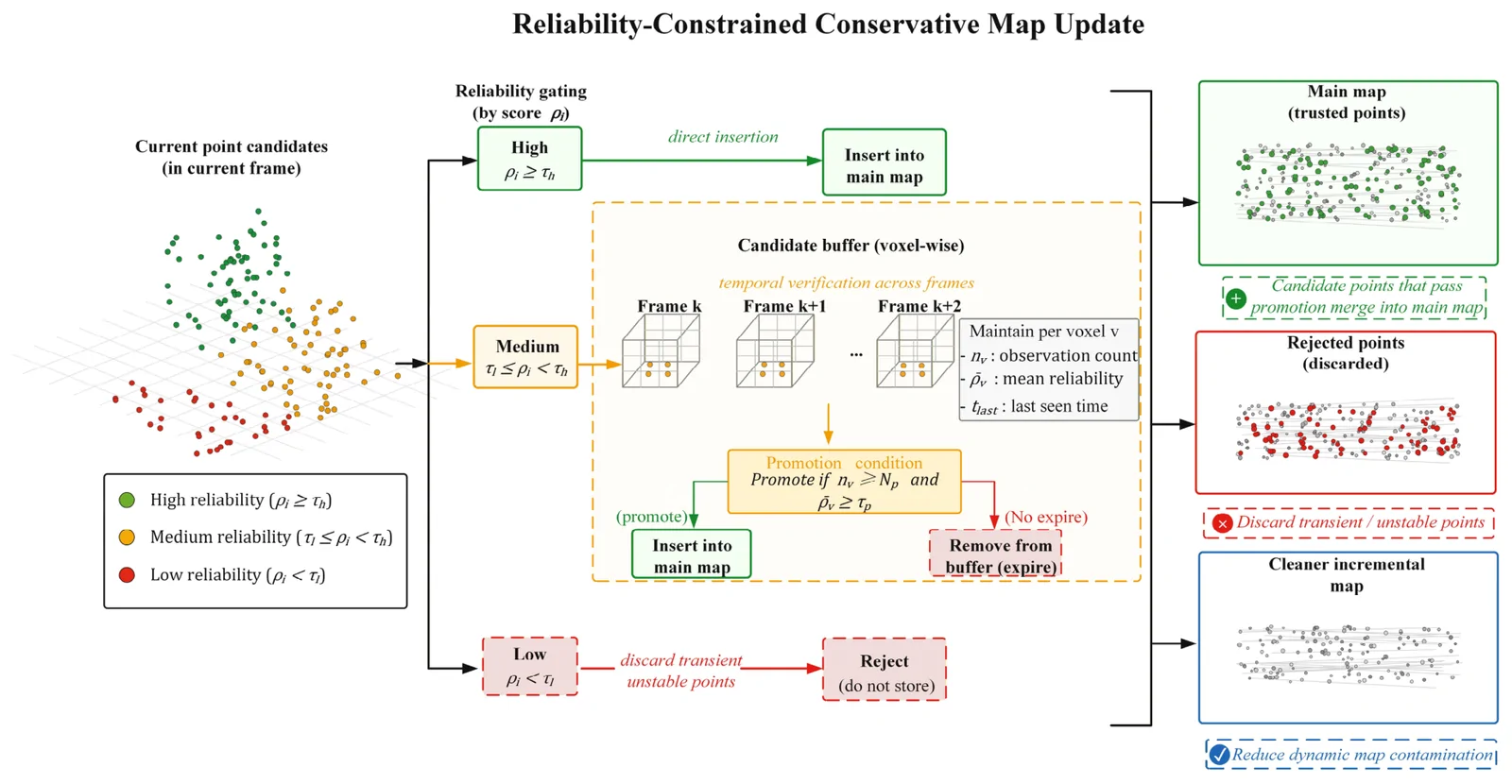
Reliability-constrained spatial-memory update strategy. High-reliability points are directly consolidated into the persistent map; medium-reliability points are temporarily stored in short-term candidate memory for multi-frame verification; and low-reliability points are rejected.

Figure 3 summarizes this reliability-constrained memory update logic. High-reliability points are consolidated directly into the persistent map, medium-reliability points are verified through short-term candidate memory, and low-reliability points are rejected. The same score first limits the instantaneous influence of uncertain residuals and then controls whether the corresponding points can become long-term spatial references, establishing a closed perception–memory loop.

### 3.4. Implementation Details and Experimental Parameters

The proposed method can be regarded as a reliability-regulated perception layer and a spatial-memory maintenance layer within a tightly coupled direct LIO system. Its objective is not to replace the original Fast-LIO2 front end, but to regulate how each point-to-map observation is used after association. Residual statistics are calculated after point-to-map association; local covariance eigenvalues are obtained from the same neighboring map points used for plane fitting; and temporal statistics are maintained at the voxel level to avoid expensive point-level history management. In this paper, +Reliability denotes Fast-LIO2 augmented with multi-cue reliability estimation and reliability-gated state update, whereas +Reliability+Map further introduces memory-gated consolidation through the candidate buffer.

To avoid conflating online statistics, derived quantities, and independently selected hyperparameters, the implementation quantities are reorganized according to their roles. They are divided into four categories: online data-dependent statistics, core scale coefficients, derived decision quantities, and engineering settings. The residual scale, geometric uncertainty, and temporal variance are calculated online; the low, high, and promotion thresholds are derived from a single decision-band parameter; and the voxel size and maximum candidate lifetime serve spatial-resolution and bounded-buffer requirements. Consequently, only the core scale coefficients and the decision-band half-width constitute independent algorithmic parameters. The main parameter settings used in the experiments are summarized in Table 1.

The nominal parameter values are fixed before evaluation and are not retuned for individual sequences. The choice of κ_r_ = 2.3849 follows the classical tuning constant of the Cauchy (Lorentzian) M-estimator, which provides approximately 95% asymptotic efficiency under a standard normal inlier distribution [34]. Because the Cauchy scale is defined as c = κ_r_σ_r_ and σ_r_ is estimated online from the current residual distribution using MAD, κ_r_ specifies a dimensionless relative attenuation scale rather than an absolute residual threshold; in particular, an observation with |r_i_| = c receives a residual reliability of 0.5, while larger deviations are progressively suppressed. For the geometric term, η_i_ = λ_1_/(λ_1_ + λ_2_ + λ_3_ + ε), together with λ_1_ ≤ λ_2_ ≤ λ_3_, implies that η_i_ lies approximately within [0, 1/3]. Accordingly, σg = 0.3 is adopted as a nominal engineering scale of the same order as the feasible range of η_i_, so that the exponential mapping provides gradual, non-saturated attenuation over geometrically uncertain planar neighborhoods rather than behaving as an abrupt geometric gate. The temporal scale σ_t_^2^ = 0.01 and the decision-band half-width Δτ = 0.15 are likewise kept fixed for all sequences. Section 4.3.5 explicitly perturbs κ_r_, σg, σ_t_^2^, and Δτ over broad ranges. Notably, several non-nominal settings yield slightly lower RMSE than the nominal point, confirming that the reported operating point was not selected by minimizing the evaluation-sequence RMSE and that the method does not rely on a narrowly tuned parameter setting.

## 4. Experiments and Results

### 4.1. Experimental Setup

Fast-LIO2 is selected as the baseline because it is a representative direct point-to-map LIO system with efficient incremental mapping capability. Three internal configurations are evaluated: Fast-LIO2, +Reliability, and +Reliability+Map. +Reliability adds the multi-cue reliability model and reliability-gated state update, whereas +Reliability+Map additionally enables candidate-memory buffering and reliability-constrained persistent-map admission. Dynamic-LIO [35] is included as an external dynamic-scene LIO baseline using its public implementation and recommended configuration. Dynamic-LIO was selected as the external comparator because it is a recent LiDAR–inertial odometry method specifically designed for dynamic driving environments, uses the same LiDAR–IMU sensing modality as the proposed framework, and provides a reproducible implementation. This enables all methods to be evaluated on identical input segments using the same reference trajectories and trajectory-error pipeline, thereby avoiding additional confounding factors caused by different sensing modalities or offline map-cleaning protocols. All methods use the same input segments, initial conditions, reference trajectories, and trajectory-error evaluation pipeline, and no parameter is retuned for an individual test sequence.

Unless otherwise stated, each quantitative configuration is repeated three times, and the tables report mean ± standard deviation or means derived from the three repeated runs. The repeated runs are used to quantify run-to-run variability, while the additional public sequences are used to assess cross-sequence robustness. Runtime measurements are performed on the same computing platform under identical software and process settings.

All experiments were conducted on a workstation equipped with an Intel Core i7-13650HX processor and 16 GB of RAM, running Ubuntu 20.04 and ROS Noetic. The proposed framework and all compared methods were executed without GPU acceleration. Runtime measurements were performed on the same computing platform under identical software and process settings to ensure a fair computational comparison.

### 4.2. Test Scenes and Evaluation Metrics

The experiments include two normal public sequences, two moderately disturbed UrbanNav sequences, two highly disturbed UrbanNav sequences, and a self-collected dynamic sequence. M2DGR street_03 is used for representative normal-scene visualization, while street_05 provides an additional normal-scene side-effect test. The representative dynamic sequences are used for trajectory visualization, whereas the additional Deep-Urban-1 and Harsh-Urban-1 sequences extend the quantitative evaluation beyond a single sequence at each dynamic level. UrbanNav-HK Medium-Urban-1 and Tunnel-1 segments are additionally used for map-quality and sensitivity analyses.

Localization accuracy is primarily evaluated using the root mean square error (RMSE), which is adopted as the principal metric for cross-sequence comparison, external benchmarking, and ablation analysis. Mean absolute error (MAE) and maximum translation error are additionally reported for the representative sequences in Section 4.3.1 to characterize average and worst-case localization behavior without overloading the cross-sequence tables. Persistent-map quality is evaluated separately in Section 4.3.4 using future-frame point-to-map RMSE, the 95th-percentile residual (P95), consistent correspondence ratio at 0.20 m (CCR@0.20), and geometric coverage. These complementary metrics distinguish localization accuracy from the ability of the persistent map to provide stable geometric support for future observations.

Public reference trajectories and the RTK reference for the self-collected sequence are transformed to a local ENU frame. Reference positions are interpolated to the estimator timestamps before translation-error computation. The normal-scene representative visualization uses the first 50 s, whereas the representative dynamic and self-collected visualizations use 90 s segments. The same evaluation windows and alignment rules are used for all methods compared on a given sequence. The evaluated datasets and corresponding scene information are listed in Table 2.

The disturbance-level labels are used as descriptive scene categories rather than as a universal dynamic-point threshold. They reflect the dataset scene characteristics and the observed persistence of moving-object interference within the evaluated segments. The self-collected Majiagou sequence contains sustained urban traffic interference and is therefore treated as a highly dynamic field scene.

The self-collected dynamic scene was recorded on 15 May 2026 on urban roads around Majiagou, Nangang District, Harbin, China. It was collected by a wheeled unmanned vehicle during the evening rush hour and represents a highly dynamic urban traffic scene. All sensors were calibrated and timestamped during data acquisition, and the coordinate systems follow the right-hand rule.

**Figure 4:**
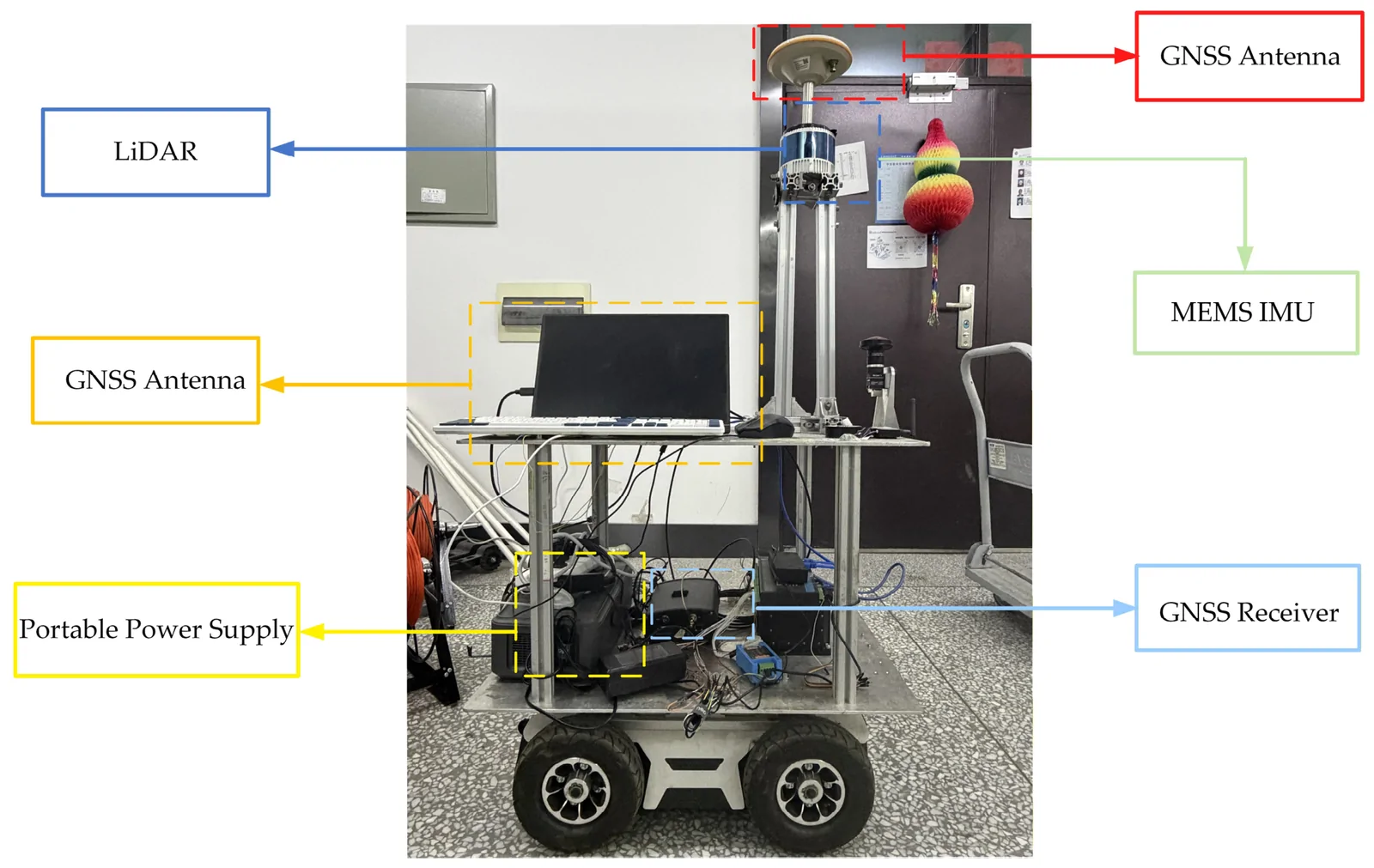
Embodied wheeled-robot experimental platform.

The wheeled robot experimental platform is shown in Figure 4.The core sensor configuration of the platform is as follows:

- LiDAR: Leishen Intelligent C32 mechanical LiDAR, 32 channels, 10 Hz, 150 m maximum range, ±2 cm ranging accuracy, and −16° to +15° vertical field of view.
- IMU: Honeywell HGuide i300 industrial-grade MEMS-IMU, 100 Hz, 5°/h gyroscope bias instability, and 30 μg accelerometer bias.
- Reference system: Shanghai Huace P3-DU BeiDou dual-antenna RTK receiver, 20 Hz output, 1.0 cm + 1 ppm horizontal accuracy, 1.5 cm + 1 ppm vertical accuracy, heading accuracy below 0.2°, and fixed-solution ratio above 95%.

The self-collected sequence was recorded in an urban traffic environment during the evening rush hour. The average traffic flow was approximately 35–45 vehicles per minute, and the estimated proportion of dynamic LiDAR points was approximately 18–22%. The scene therefore contains persistent interference from moving vehicles, temporary occlusions, and changing point-to-map correspondences, and is categorized as a highly dynamic environment according to the criteria defined above.

**Figure 5:**
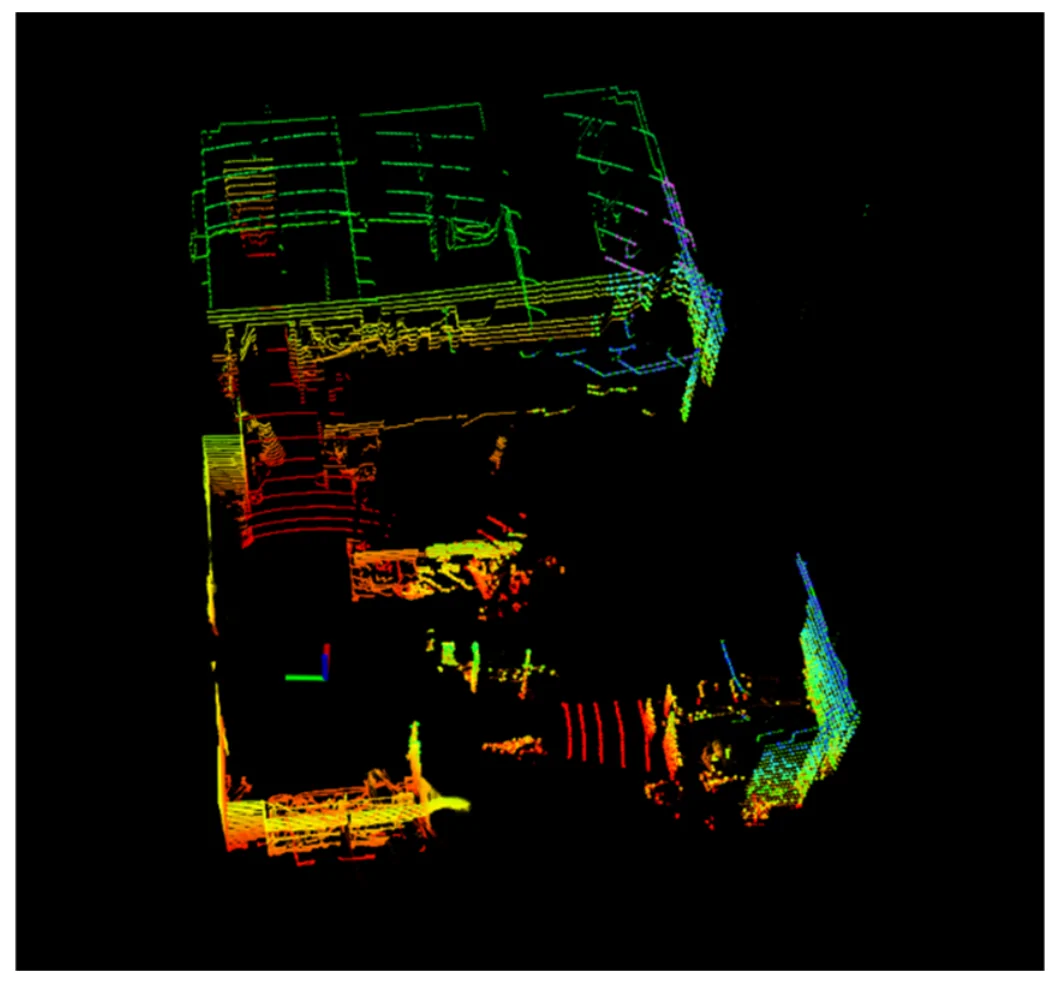
Calibration process.

Before tightly coupled LiDAR–IMU processing, spatial and temporal calibration between the LiDAR and IMU was performed using the open-source LI-Init toolbox [31]. The calibration estimates the relative rotation and translation between the LiDAR and IMU coordinate systems, together with the IMU-to-LiDAR time offset and the initial inertial quantities required for system initialization. These calibration results are used to transform LiDAR measurements into the unified estimation frame and to compensate for inter-sensor time delay before scan undistortion and ESIKF-based fusion. Figure 5 illustrates the calibration result, while the estimated rotation, translation, inertial biases, gravity vector, and time offset are reported in Table 3.

### 4.3. Experimental Results and Analysis

The experimental evaluation is organized from six complementary perspectives: representative localization behavior, cross-sequence robustness and comparison with Dynamic-LIO, module ablation, persistent-map quality, hyperparameter sensitivity, and computational efficiency.

#### 4.3.1. Representative Localization Results

**Figure 6:**
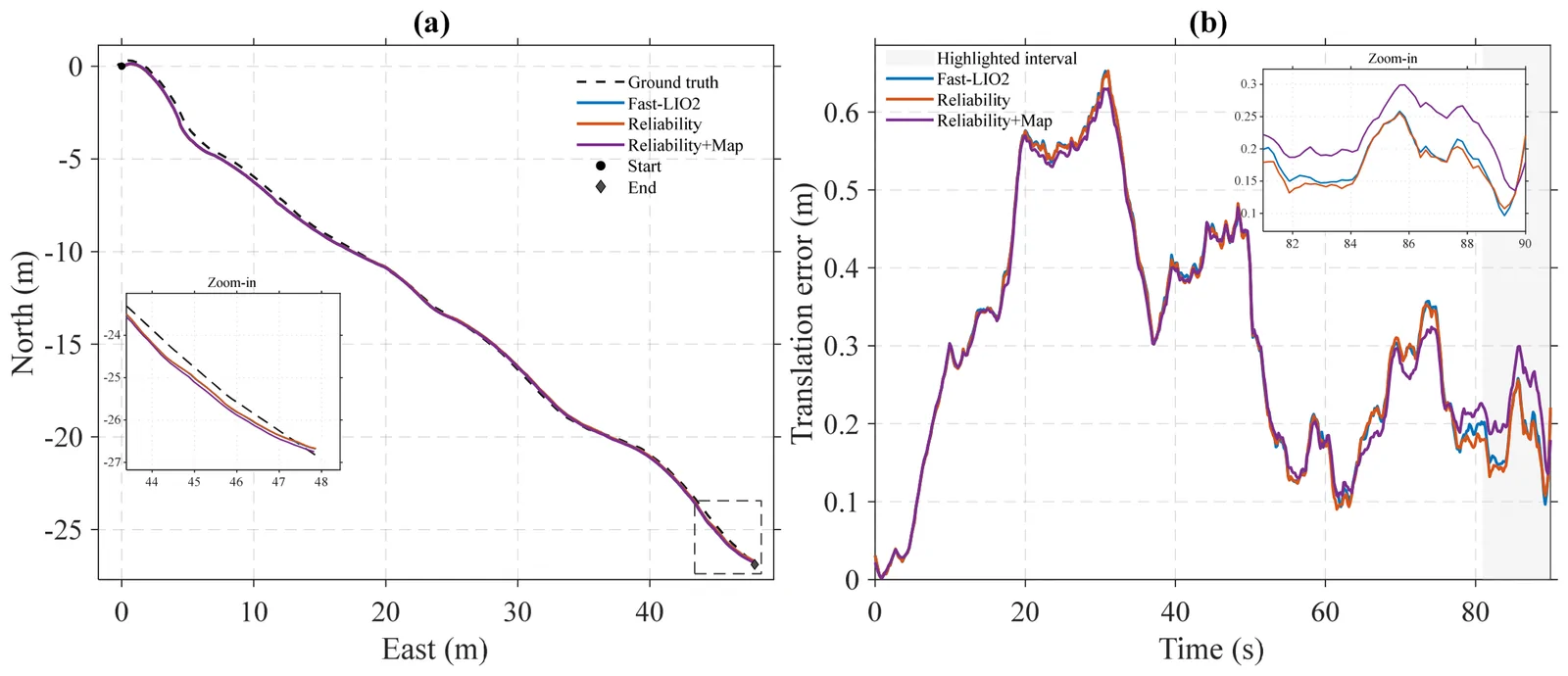
Localization results in the representative normal sequence (M2DGR street_03). (**a**) Trajectory comparison among ground truth, Fast-LIO2, +Reliability, and +Reliability+Map. (**b**) Translation error comparison among Fast-LIO2, +Reliability, and +Reliability+Map. The plotted curves correspond to a representative run.

Figure 6a shows that all three LIO configurations remain close to the reference trajectory in the normal scene, confirming that the proposed reliability mechanisms do not introduce visible trajectory distortion under predominantly stable geometry. Across three repeated runs, the RMSE, MAE, and maximum error of Fast-LIO2 are 0.3482 ± 0.0052 m, 0.3043 ± 0.0041 m, and 0.6615 ± 0.0087 m, respectively. +Reliability yields 0.3468 ± 0.0044 m, 0.3040 ± 0.0035 m, and 0.6601 ± 0.0079 m, while +Reliability+Map yields 0.3454 ± 0.0051 m, 0.2995 ± 0.0043 m, and 0.6598 ± 0.0092 m. The corresponding RMSE reductions relative to Fast-LIO2 are 0.40% and 0.80%, respectively.

Figure 6b further shows that the translation-error curves remain closely overlapped for most of the interval. Both proposed configurations remain close to Fast-LIO2 in this stable sequence. An additional normal-scene test on M2DGR street_05 yields RMSE values of 0.0956 ± 0.0017 m, 0.0929 ± 0.0012 m, and 0.0917 ± 0.0013 m for Fast-LIO2, +Reliability, and +Reliability+Map, respectively, corresponding to RMSE reductions of 2.82% and 4.08%. The two normal sequences therefore indicate that reliability regulation and map admission do not degrade localization accuracy under predominantly static conditions.

**Figure 7:**
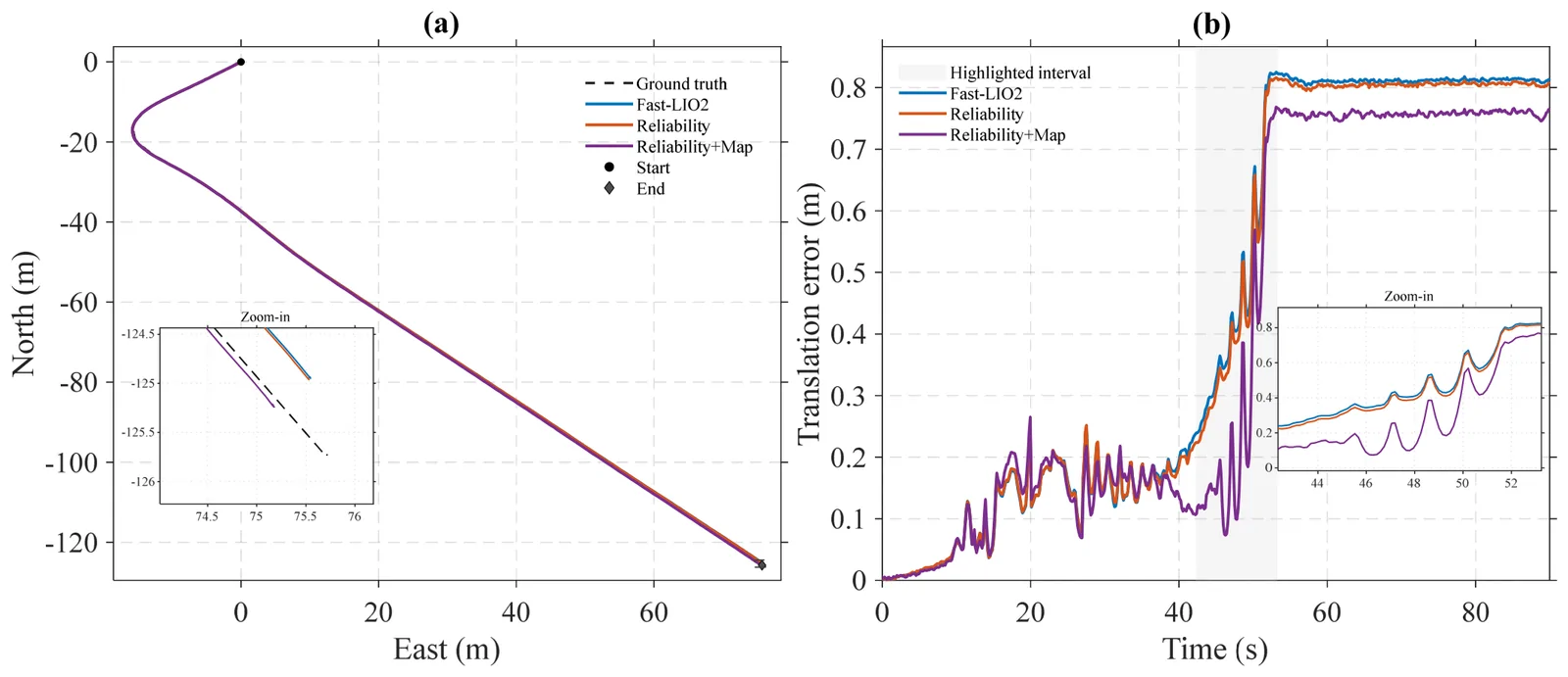
Localization results in the moderately dynamic scene. (**a**) Trajectory comparison among ground truth, Fast-LIO2, +Reliability, and +Reliability+Map. (**b**) Translation error comparison among the three methods. The plotted curves correspond to a representative run.

Figure 7a shows the representative trajectory behavior in the moderately dynamic scene. The trajectories remain globally similar, but the proposed configurations reduce the accumulated separation from the reference during the later part of the segment. Across three runs, the RMSE decreases from 0.5893 ± 0.0085 m for Fast-LIO2 to 0.5455 ± 0.0077 m with +Reliability and 0.5063 ± 0.0094 m with +Reliability+Map, corresponding to reductions of 7.43% and 14.08%, respectively. The associated MAE values are 0.4798 ± 0.0068 m, 0.4572 ± 0.0059 m, and 0.3987 ± 0.0071 m, and the maximum errors are 0.8357 ± 0.0113 m, 0.8093 ± 0.0108 m, and 0.7498 ± 0.0121 m.

**Figure 8:**
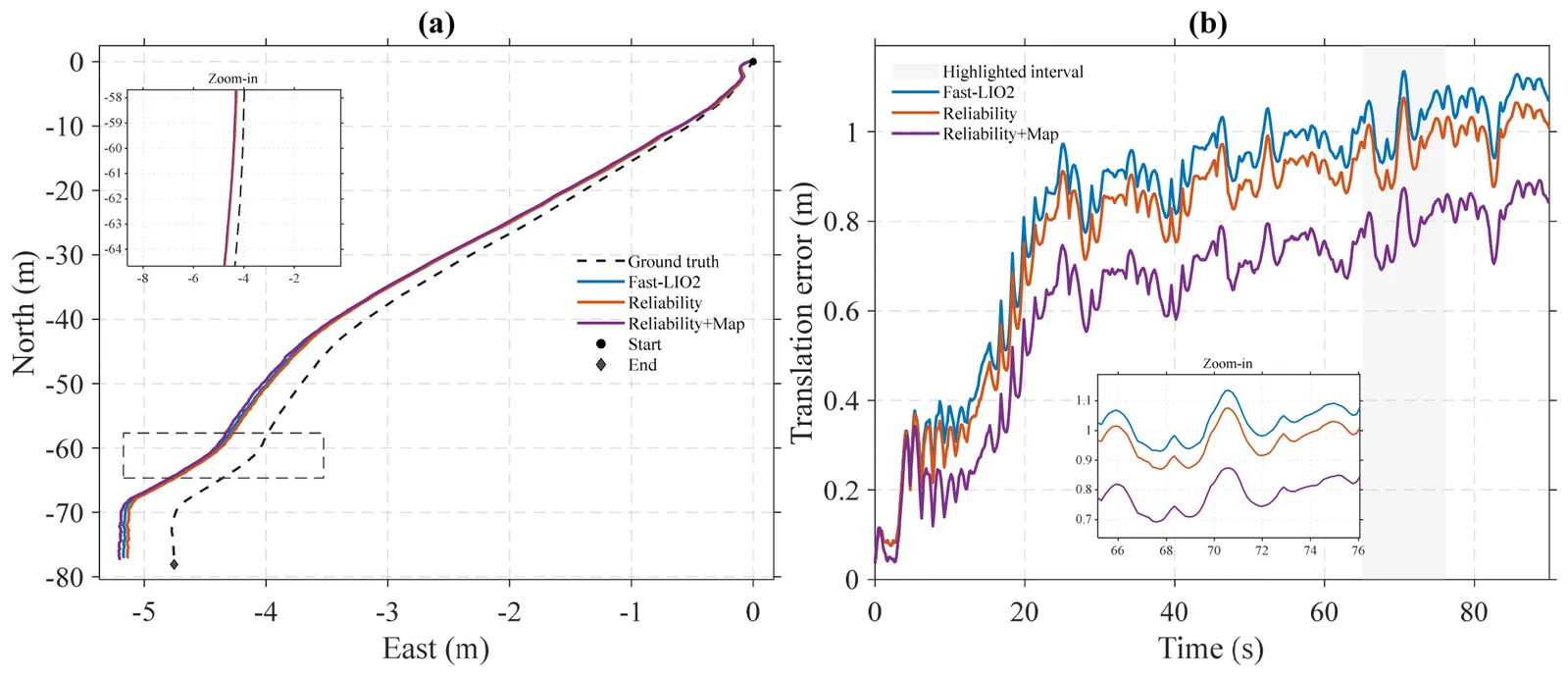
Localization results in the highly dynamic scene. (**a**) Trajectory comparison among ground truth, Fast-LIO2, +Reliability, and +Reliability+Map. (**b**) Translation error comparison among the three methods. The plotted curves correspond to a representative run.

**Figure 9:**
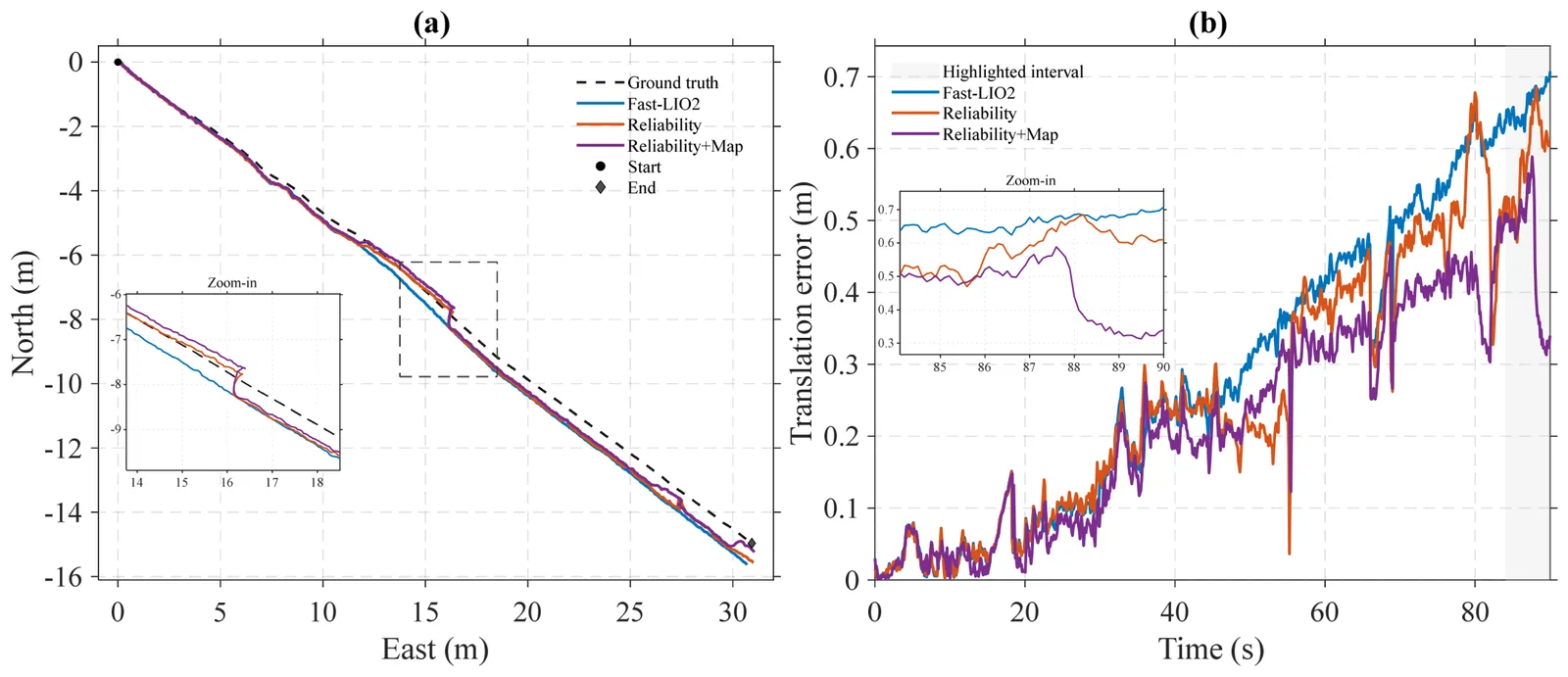
Field experimental results on self-collected dynamic data. (**a**) Trajectory comparison among ground truth, Fast-LIO2, +Reliability, and +Reliability+Map. (**b**) Translation error comparison among the three methods. The plotted curves correspond to a representative run.

Figure 7b shows that +Reliability first suppresses part of the intermittent error growth by reducing the influence of unreliable point-to-map residuals. Activating memory-gated map consolidation provides a further 7.19% RMSE reduction relative to +Reliability. The relatively small trajectory separation in Figure 7a compared with Figure 8a and Figure 9a is consistent with less persistent dynamic interference: local map corruption accumulates more slowly, so the baseline and proposed trajectories remain closer over the evaluated interval.

Figure 8a shows a progressively increasing separation between the reference trajectory and the LIO estimates in the highly dynamic scene. This behavior is consistent with persistent moving-object interference affecting both current-frame correspondences and the incremental map, so local association bias can accumulate as the trajectory proceeds. Across three runs, Fast-LIO2, +Reliability, and +Reliability+Map obtain RMSE values of 0.8751 ± 0.0153 m, 0.8153 ± 0.0136 m, and 0.6542 ± 0.0112 m, respectively. The corresponding reductions relative to Fast-LIO2 are 6.83% and 25.24%.

Figure 8b confirms that reliability weighting alone reduces part of the instantaneous error, while the full method produces a substantially larger improvement after map-history effects become important. The MAE decreases from 0.8254 ± 0.0142 m to 0.6247 ± 0.0105 m, and the maximum error decreases from 1.1283 ± 0.0184 m to 0.8939 ± 0.0149 m. Relative to +Reliability, adding map-memory regulation further reduces RMSE by 19.76%, supporting the need to prevent uncertain observations from becoming persistent references in strongly dynamic conditions.

Figure 9a presents the field experiment on the self-collected Majiagou sequence. The gradual trajectory separation is more evident than in the moderately dynamic public sequence because the field data contain sustained vehicle motion, temporary occlusions, platform vibration, and real sensor/reference alignment uncertainty. Across three repeated runs, the RMSE decreases from 0.3755 ± 0.0058 m for Fast-LIO2 to 0.3364 ± 0.0049 m with +Reliability and 0.2841 ± 0.0042 m with +Reliability+Map, corresponding to reductions of 10.41% and 24.34%.

Figure 9b shows that the full method maintains the lowest translation error over most of the later segment. The MAE decreases from 0.3086 ± 0.0047 m to 0.2353 ± 0.0034 m, and the maximum error decreases from 0.7174 ± 0.0096 m to 0.5821 ± 0.0076 m. Compared with +Reliability, the map-maintenance module provides an additional 15.55% RMSE reduction, indicating that the delayed map-contamination pathway remains important in real traffic rather than only in public benchmark data. For compact cross-sequence reporting, Table 4 therefore retains RMSE as the principal metric, while MAE and maximum error are reported here for the representative sequences and are not redundantly expanded for every additional sequence.

Table 4 extends the evaluation beyond the representative plots. The complete method reduces RMSE on every listed sequence. On the two normal M2DGR sequences, the complete method changes the RMSE only slightly, with reductions of 0.80% on street_03 and 4.08% on street_05, supporting the side-effect test under predominantly static conditions. Across the five dynamic sequences (Medium-Urban-1, Deep-Urban-1, Tunnel-1, Harsh-Urban-1, and Majiagou), the RMSE reduction relative to Fast-LIO2 ranges from 14.08% to 28.88%, with a mean relative reduction of 22.11%. +Reliability alone also improves all five dynamic sequences, showing that reliability-regulated perception contributes consistently before map-memory regulation is enabled.

#### 4.3.2. Cross-Sequence Comparison with Dynamic-LIO

To benchmark the proposed framework against an independently developed dynamic-scene LIO method, Dynamic-LIO [34] is evaluated on the five dynamic sequences using the same input segments and reference-trajectory evaluation pipeline. Table 5 reports RMSE because it provides the most compact cross-sequence comparison while the representative mean and maximum errors have already been analyzed in Section 4.3.1.

The proposed method achieves lower mean RMSE than Dynamic-LIO on all five evaluated dynamic sequences, with improvements of 6.88%, 14.85%, 7.83%, 7.49%, and 4.76%, respectively. Using the sequence-level mean RMSE values, a one-sided Wilcoxon signed-rank test gives *p* = 0.031 for +Reliability+Map versus Fast-LIO2 and *p* = 0.031 for +Reliability+Map versus Dynamic-LIO. The non-parametric test is used because only five heterogeneous dynamic sequences are available. These results support consistent cross-sequence improvement without assuming normally distributed sequence-level errors.

#### 4.3.3. Ablation Analysis

To analyze the relative contributions of reliability-regulated perception and memory-gated map consolidation, ablation experiments are conducted on three representative dynamic sequences with different disturbance levels. The purpose of this experiment is to distinguish the effect of improving the current state update from the additional benefit obtained by constraining the long-term evolution of the incremental map. In the normal scene, most point-to-map correspondences are generated from stable static structures, and the reliability scores therefore remain predominantly high. Under such conditions, the proposed modules are expected to produce only limited changes in localization accuracy, and the normal-scene evaluation mainly serves as a side-effect test to verify that reliable static observations are not unnecessarily suppressed or rejected. As the level and persistence of dynamic disturbance increase, however, unreliable correspondences occur more frequently and are more likely to influence both the current ESIKF correction and subsequent map maintenance. The differences among Fast-LIO2, +Reliability, and +Reliability+Map therefore become more observable in the dynamic sequences. Specifically, the +Reliability configuration isolates the contribution of continuous reliability weighting to instantaneous state estimation, whereas the +Reliability+Map configuration further evaluates whether preventing uncertain observations from being directly consolidated into the persistent map can reduce the delayed accumulation of map-induced errors. This progressive comparison makes it possible to determine whether reliability regulation should remain confined to the current perceptual update or should also govern the formation and maintenance of persistent spatial memory.

**Figure 10:**
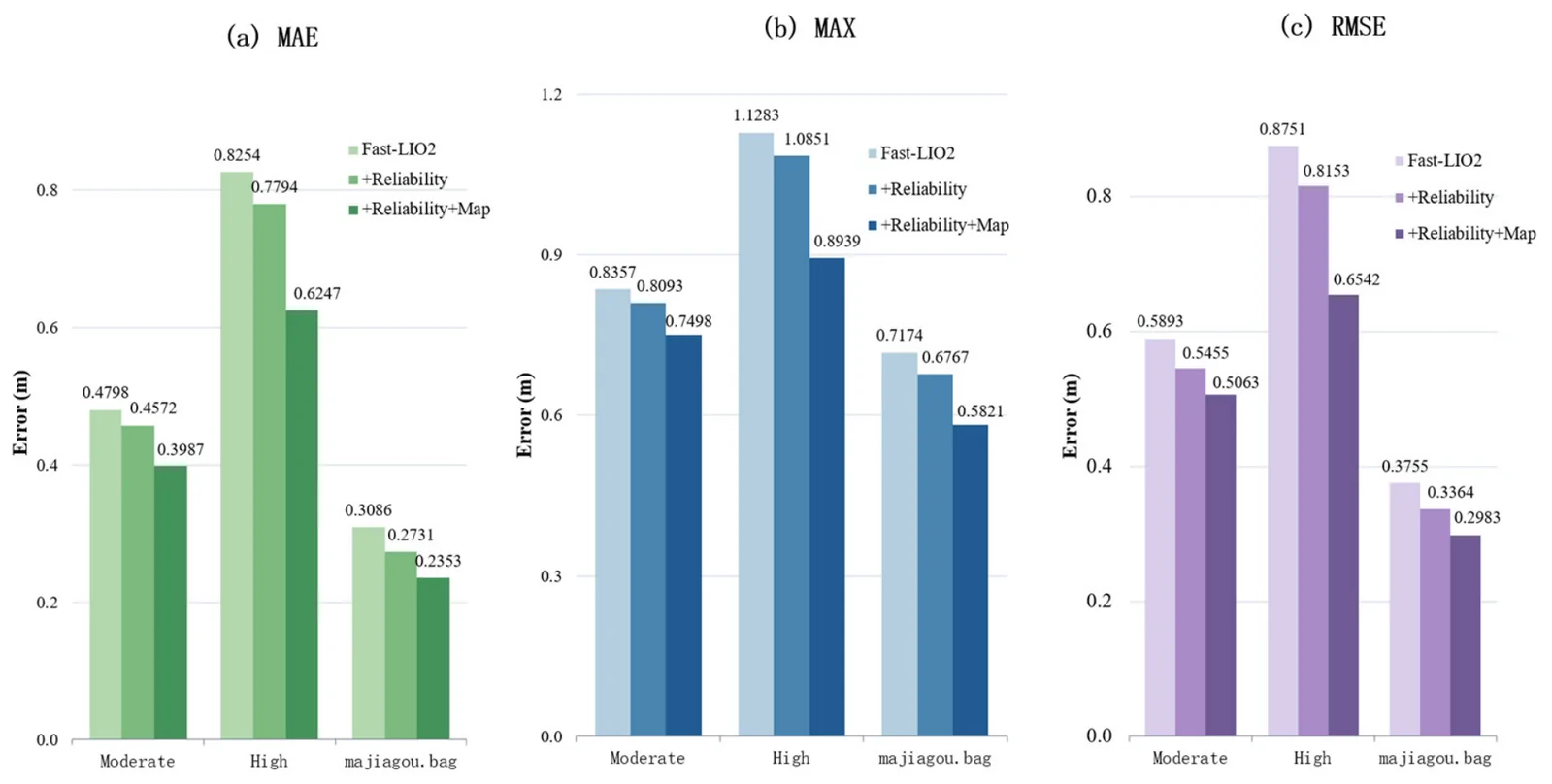
Ablation of reliability-regulated perception and memory-gated map consolidation on three representative dynamic sequences. The bars show the mean results over three repeated runs. (**a**) MAE. (**b**) Maximum translation error. (**c**) RMSE.

Figure 10 further compares the contributions of reliability-regulated perception and memory-gated map consolidation in terms of MAE, maximum error, and RMSE. As shown in Figure 10a–c, the +Reliability configuration consistently reduces the overall localization error relative to Fast-LIO2, indicating that continuous reliability weighting effectively suppresses the instantaneous influence of unreliable point-to-map observations. After enabling the map-maintenance module, +Reliability+Map achieves further error reductions across all three representative dynamic scenes. This additional improvement is particularly evident in Tunnel-1 and Majiagou, where persistent moving-object interference is more likely to contaminate the incremental map and affect subsequent point-to-map associations. In terms of RMSE, +Reliability reduces the error by 7.43%, 6.83%, and 10.41% on Medium-Urban-1, Tunnel-1, and Majiagou, respectively, whereas the complete configuration increases the reductions to 14.08%, 25.24%, and 24.34%. Relative to +Reliability alone, memory-gated map consolidation provides additional RMSE reductions of 7.19%, 19.76%, and 15.55%, respectively. The consistent trends in MAE, maximum error, and RMSE indicate that the two proposed modules play complementary roles: reliability weighting mainly limits current-frame estimation errors, while selective map admission prevents unreliable observations from becoming persistent references and thereby reduces delayed error propagation in strongly dynamic environments.

#### 4.3.4. Persistent-Map Quality Evaluation

The trajectory results demonstrate improved localization, but the proposed motivation also concerns whether unreliable observations become persistent map references. A frozen-map future-frame consistency experiment is therefore conducted on UrbanNav-HK Medium-Urban-1, UrbanNav-HK Tunnel-1, and Majiagou. For each run, the first 60 s are used for map construction; the map is then frozen, and LiDAR observations from 60 to 90 s are transformed using the common reference pose and evaluated against the frozen map. The same neighborhood search, plane-validity criteria, sampling rule, and consistency threshold are used for all methods.

For each validation point *p_i_W*, which is transformed into the world frame using the common reference pose, a local neighborhood is searched in the frozen map and a plane is fitted using the same correspondence-search and plane-validity criteria for all compared methods. For each valid correspondence i ∈ 𝒱, the absolute point-to-plane residual is defined as

(22) $$e_{i}=\left|n_{i}^{T}p_{i}^{W}+d_{i}\right|$$

where *n_i_* and *d_i_* denote the unit normal vector and offset of the locally fitted map plane, respectively. The set 𝒱 contains all validation points for which a valid point-to-map correspondence can be established under the fixed evaluation criteria. The same neighborhood search, plane-validity criterion, sampling procedure, and residual-computation rule are applied to all methods to ensure a consistent evaluation protocol.

Based on the valid correspondence set, four complementary metrics are used to characterize frozen-map consistency and geometric usability. The future-frame point-to-map RMSE is defined as

(23) $$E_{map}=\sqrt{\frac{1}{\left|V\right|}\sum_{i\in V}e_{i}^{2}}$$

which measures the overall geometric inconsistency between future-frame observations and the frozen persistent map. The P95 metric is defined as the 95th percentile of the residual set {*e_i_*}*_i_* ∈ 𝒱, thereby characterizing the upper tail of the point-to-map residual distribution while being less sensitive to a single extreme residual than the maximum error. Lower values of *E_map_* and P95 indicate stronger geometric consistency.

To further evaluate the proportion of geometrically consistent correspondences, the consistent correspondence ratio (CCR) is defined as

(24) $${CCR}_{\delta }=\frac{\sum_{i\in V}1\left(e_{i}\leq \delta \right)}{\left|V\right|}\times 100\%$$

where 1(·) is the indicator function and the consistency threshold is fixed at δ = 0.20 m for all methods and evaluated datasets. Accordingly, CCR@0.20 represents the proportion of valid correspondences whose point-to-plane residual does not exceed 0.20 m. In addition, geometric coverage is defined as

(25) $$Coverage=\frac{\left|V\right|}{N_{val}}\times 100\%$$

where *N_val_* denotes the total number of validation points entering the evaluation procedure. Coverage therefore measures the proportion of future observations that can obtain valid geometric support from the frozen map.

Because the valid correspondence set is map-dependent, the residual-based metrics are interpreted jointly with CCR and coverage rather than in isolation. In particular, coverage is reported to avoid attributing a lower point-to-map RMSE to improved map quality when the reduction could otherwise result from retaining only a small subset of easy-to-match validation points. Therefore, lower map RMSE and P95, together with higher CCR and coverage, indicate that the persistent map provides both stronger geometric consistency and more usable support for subsequent observations. As shown in Table 6, lower map RMSE and P95, together with higher CCR and coverage, indicate that the persistent map provides both stronger geometric consistency and more usable support for subsequent observations.

Relative to Fast-LIO2, +Reliability+Map reduces map RMSE by 7.77%, 12.83%, and 15.71% on Medium-Urban-1, Tunnel-1, and Majiagou, respectively. The corresponding P95 reductions are 8.20%, 13.31%, and 16.20%, while CCR increases by 5.13, 5.41, and 8.92 percentage points. Coverage also increases by 2.42, 3.41, and 3.92 percentage points. Because coverage measures the proportion of future observations that obtain valid geometric support rather than raw map-point density, its increase indicates that the more selective admission rule yields a more usable persistent map rather than merely discarding difficult correspondences.

#### 4.3.5. Hyperparameter Sensitivity Analysis

To test whether the method depends on a narrowly tuned operating point, a one-factor-at-a-time sensitivity analysis is performed for κ_r_, σg, σ_t_^2^, and Δτ on Medium-Urban-1, Tunnel-1, and Majiagou. Each parameter configuration is repeated three times, while all other parameters are fixed at their nominal values. The tested ranges are κ_r_ ∈ [1.5, 3.5], σg ∈ [0.20, 0.40], σ_t_^2^ ∈ [0.005, 0.020], and Δτ ∈ [0.10, 0.20]. The analysis is intended to assess stability around the nominal operating point rather than to search for a sequence-specific optimum.

**Figure 11:**
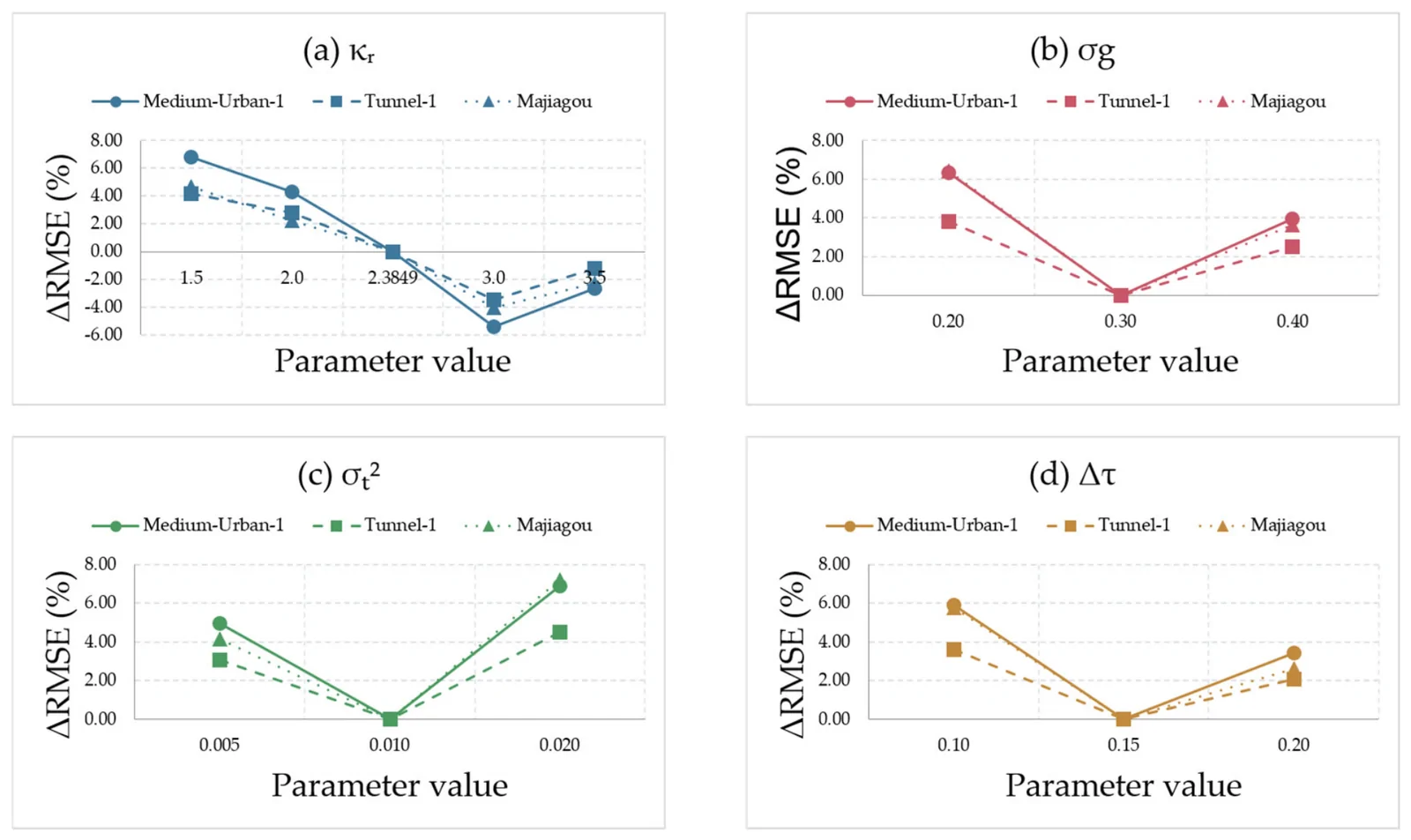
Sensitivity of localization RMSE to the four principal hyperparameters. Each point is the mean of three runs and is expressed relative to the nominal configuration.

Figure 11 shows smooth performance changes over the investigated ranges without estimator failure or abrupt degradation. In particular, κ_r_ = 3.0 produces slightly lower RMSE than the nominal κ_r_ = 2.3849 on all three sequences, which further indicates that the nominal coefficient was not selected by minimizing the test-sequence RMSE. The purpose of the nominal value is therefore to provide a fixed operating point rather than a claimed global optimum.

To verify the robustness of the proposed method, we conduct sensitivity analysis on core parameters. The maximum absolute RMSE deviation from the nominal configuration for each parameter is presented in Table 7, with detailed analysis provided in the subsequent section.

Across the three sequences, the largest mean RMSE deviation from the nominal configuration is 7.19%, observed for σ_t_^2^ on Majiagou. The run-to-run standard deviations over all sensitivity configurations are 0.0033–0.0072 m for Medium-Urban-1, 0.0055–0.0102 m for Tunnel-1, and 0.0019–0.0041 m for Majiagou. These results support limited sensitivity to moderate parameter variation rather than dependence on a sharply tuned operating point.

#### 4.3.6. Runtime and Computational Analysis

Runtime is measured as the average processing time per LiDAR scan on three datasets. Each configuration is repeated three times under identical input and process settings, and Table 8 reports mean ± standard deviation together with the overhead relative to Fast-LIO2. The runtime measurements use the computing platform specified in Section 4.1.

As shown in Table 8, +Reliability increases the mean processing time from 11.31 to 14.22 ms on Medium-Urban-1, from 10.43 to 13.60 ms on Tunnel-1, and from 14.61 to 17.36 ms on Majiagou. With map-memory regulation enabled, the corresponding times are 16.57, 15.43, and 18.69 ms. The relative overhead of the full method is therefore 46.51%, 47.94%, and 27.93%, respectively.

The repeated timing measurements remove the counter-intuitive ordering observed in the previous single-run result: +Reliability+Map is consistently slower than +Reliability on all three datasets, as expected from its additional candidate-buffer and map-admission operations. The small standard deviations indicate stable runtime behavior across repeated runs.

Despite the added computation, the maximum measured mean runtime is 18.69 ms per scan, well below the 100 ms inter-frame interval of a 10 Hz LiDAR. The proposed framework therefore introduces measurable computational overhead but maintains real-time operation with substantial timing margin on the evaluated platform.

## 5. Conclusions

This paper presents a functionally bio-inspired perception–memory coupling framework for robust LiDAR–inertial localization of intelligent mobile robots. Residual consistency, local geometric quality, and voxel-level temporal stability are integrated into a point-wise reliability score that regulates both the ESIKF information contribution and persistent map admission. Ambiguous observations are retained in short-term candidate memory, while repeatedly supported evidence is consolidated into the incremental map.

Repeated cross-sequence experiments show that the complete +Reliability+Map method maintains comparable or slightly improved accuracy on the two normal M2DGR sequences, with RMSE reductions of 0.80% and 4.08%, and reduces localization RMSE by 14.08–28.88% relative to Fast-LIO2 across five dynamic sequences. It also achieves lower mean RMSE than Dynamic-LIO on all five evaluated dynamic sequences. The frozen-map experiment provides direct map-level evidence: map RMSE decreases by 7.77–15.71%, accompanied by lower P95 residuals and higher CCR and coverage. The sensitivity study shows that the maximum mean RMSE deviation remains below 7.2% over the investigated parameter ranges, and the runtime analysis confirms a maximum mean processing time of 18.69 ms per scan.

From the perspective of bionic intelligence, these results support the functional principle that uncertain perceptual evidence should have limited instantaneous influence and should require additional support before becoming a persistent environmental reference. The proposed framework remains an engineering abstraction rather than a biologically faithful neural model and requires neither semantic labels nor training data. Future work will investigate adaptive reliability calibration, bounded candidate-memory forgetting, longer-duration map-quality evaluation, and complementary semantic, motion-consistency, and occupancy evidence.

## Figures and Tables

**Table 1:** Main parameter settings of the proposed perception–memory framework.

Parameter	Symbol/Implementation Name	Setting or Determination Rule
Residual attenuation coefficient	κ_r_	2.3849; fixed for all sequences
Geometric attenuation scale	σ_g_	0.3; fixed for all sequences
Temporal reliability variance scale	σ_t_^2^	0.01; fixed for all sequences
Reliability decision band	Δτ	0.15; τ_l_ = 0.35, τ_h_ = 0.65, and τ_p_ = 0.50
Candidate promotion persistence	N_p_	2 consecutive scan observations
Candidate voxel size	candidate voxel size	0.5 m
Maximum candidate lifetime	candidate max age	3.0 s

**Table 2:** Dataset and scene information.

Scene	Dataset	Sequence/File	Disturbance Level	Reference
Normal scene	M2DGR [3]	street_03	Low	Dataset reference
Normal scene	M2DGR [3]	street_05	Low	Dataset reference
Moderately dynamic	UrbanNav [4]	Medium-Urban-1	Moderate	Dataset reference
Moderately dynamic	UrbanNav [4]	Deep-Urban-1	Moderate	Dataset reference
Highly dynamic	UrbanNav [4]	Tunnel-1	High	Dataset reference
Highly dynamic	UrbanNav [4]	Harsh-Urban-1	High	Dataset reference
Self-collected dynamic	Self-collected	majiagou.bag	High	RTK

**Table 3:** LiDAR–IMU calibration and initialization results.

Parameter Type	X Axis	Y Axis	Z Axis
Rotation LiDAR → IMU (°)	−0.063135	−0.221756	−0.735634
Translation LiDAR → IMU (m)	0.064654	−0.043243	0.246635
Gyroscope bias (rad/s)	0.000345	0.001156	0.000767
Accelerometer bias (m/s^2^)	−0.008452	−0.001563	−0.006344
Gravity vector in the world frame (m/s^2^)	−0.039631	0.045457	−9.835635
Time delay IMU → LiDAR (s)	0.235775		

**Table 4:** Cross-sequence localization RMSE of the three internal configurations (mean ± std, n = 3).

Sequence	Fast-LIO2 RMSE (m)	+Reliability RMSE (m)	+Reliability+Map RMSE (m)	Full Reduction (%)
M2DGR street_03	0.3482 ± 0.0052	0.3468 ± 0.0044	0.3454 ± 0.0051	0.80%
M2DGR street_05	0.0956 ± 0.0017	0.0929 ± 0.0012	0.0917 ± 0.0013	4.08%
Medium-Urban-1	0.5893 ± 0.0085	0.5455 ± 0.0077	0.5063 ± 0.0094	14.08%
Deep-Urban-1	1.3328 ± 0.0221	1.2567 ± 0.0214	1.0932 ± 0.0186	17.98%
Tunnel-1	0.8751 ± 0.0153	0.8153 ± 0.0136	0.6542 ± 0.0112	25.24%
Harsh-Urban-1	2.5323 ± 0.0448	2.3740 ± 0.0419	1.8009 ± 0.0314	28.88%
majiagou.bag	0.3755 ± 0.0058	0.3364 ± 0.0049	0.2841 ± 0.0042	24.34%

**Table 5:** RMSE comparison with Dynamic-LIO on five dynamic sequences (mean ± std, n = 3).

Sequence	Fast-LIO2 (m)	Dynamic-LIO (m)	Proposed (m)	vs. Fast-LIO2	vs. Dynamic-LIO
Medium-Urban-1	0.5893 ± 0.0085	0.5437 ± 0.0073	0.5063 ± 0.0094	14.08%	6.88%
Deep-Urban-1	1.3328 ± 0.0221	1.2839 ± 0.0209	1.0932 ± 0.0186	17.98%	14.85%
Tunnel-1	0.8751 ± 0.0153	0.7098 ± 0.0124	0.6542 ± 0.0112	25.24%	7.83%
Harsh-Urban-1	2.5323 ± 0.0448	1.9467 ± 0.0341	1.8009 ± 0.0314	28.88%	7.49%
majiagou.bag	0.3755 ± 0.0058	0.2983 ± 0.0045	0.2841 ± 0.0042	24.34%	4.76%

**Table 6:** Quantitative comparison of persistent-map quality (mean ± std, n = 3).

Dataset	Method	Map RMSE (m)	P95 (m)	CCR@0.20 (%)	Coverage (%)
Medium-Urban-1	Fast-LIO2	0.2510 ± 0.0021	0.5741 ± 0.0045	68.69 ± 0.63	54.47 ± 0.57
	+Reliability	0.2402 ± 0.0018	0.5483 ± 0.0046	71.15 ± 0.56	55.62 ± 0.54
	+Reliability+Map	0.2315 ± 0.0021	0.5270 ± 0.0038	73.82 ± 0.60	56.89 ± 0.64
Tunnel-1	Fast-LIO2	0.3718 ± 0.0027	0.7034 ± 0.0060	37.50 ± 0.45	58.27 ± 0.55
	+Reliability	0.3476 ± 0.0018	0.6542 ± 0.0050	39.98 ± 0.43	59.85 ± 0.51
	+Reliability+Map	0.3241 ± 0.0029	0.6098 ± 0.0057	42.91 ± 0.41	61.68 ± 0.64
Majiagou	Fast-LIO2	0.3221 ± 0.0032	0.7356 ± 0.0060	61.66 ± 0.60	55.50 ± 0.49
	+Reliability	0.2968 ± 0.0027	0.6729 ± 0.0050	65.73 ± 0.55	57.31 ± 0.58
	+Reliability+Map	0.2715 ± 0.0023	0.6164 ± 0.0059	70.58 ± 0.54	59.42 ± 0.55

**Table 7:** Maximum absolute RMSE deviation from the nominal configuration.

Parameter	Nominal	Tested Range	Medium-Urban-1 Max |ΔRMSE|	Tunnel-1 Max |ΔRMSE|	Majiagou Max |ΔRMSE|	Worst Case
κ_r_	2.3849	1.5–3.5	6.79%	4.20%	4.68%	6.79%
σg	0.30	0.20–0.40	6.32%	3.79%	6.43%	6.43%
σ_t_^2^	0.010	0.005–0.020	6.91%	4.50%	7.19%	7.19%
Δτ	0.15	0.10–0.20	5.92%	3.60%	5.79%	5.92%

**Table 8:** Runtime comparison on three datasets (mean ± std, n = 3).

Dataset	Method	Time per Scan (ms)	Relative Overhead (%)
Medium-Urban-1	Baseline	11.31 ± 0.13	—
	+Reliability	14.22 ± 0.31	25.73 ± 0.77
	+Reliability+Map	16.57 ± 0.45	46.51 ± 1.21
Tunnel-1	Baseline	10.43 ± 0.30	—
	+Reliability	13.60 ± 0.03	30.39 ± 0.94
	+Reliability+Map	15.43 ± 0.11	47.94 ± 1.38
majiagou	Baseline	14.61 ± 0.50	—
	+Reliability	17.36 ± 0.42	18.82 ± 0.63
	+Reliability+Map	18.69 ± 0.17	27.93 ± 0.98

## Data Availability

The public datasets analyzed in this paper can be accessed via their official open-source links. The self-collected point cloud dataset is available from the corresponding author upon reasonable request.
